# Study protocol for writing to heal: A culturally based brief expressive writing intervention for Chinese immigrant breast cancer survivors

**DOI:** 10.1371/journal.pone.0309138

**Published:** 2024-09-26

**Authors:** Qian Lu, Di Lun, Lenna Dawkins-Moultin, Yisheng Li, Minxing Chen, Sharon Hermes Giordano, James W. Pennebaker, Lucy Young, Carol Wang

**Affiliations:** 1 Department of Health Disparities Research, The University of Texas MD Anderson Cancer Center, Houston, Texas, United States of America; 2 Department of Biostatistics, The University of Texas MD Anderson Cancer Center, Houston, Texas, United States of America; 3 Department of Health Services Research, The University of Texas MD Anderson Cancer Center, Houston, Texas, United States of America; 4 Department of Psychology, The University of Texas at Austin, Austin, Texas, United States of America; 5 Herald Cancer Association, San Gabriel, California, United States of America; Public Library of Science, UNITED KINGDOM OF GREAT BRITAIN AND NORTHERN IRELAND

## Abstract

**Background:**

This study uses a randomized controlled trial (RCT) to test the health benefits of expressive writing that is culturally adapted for Chinese immigrant breast cancer survivors (BCSs) and to characterize how acculturation moderates the effects of expressive writing interventions.

**Methods:**

We will recruit Chinese immigrant BCSs (N = 240) diagnosed with stage 0-III breast cancer and within 5 years of completion of primary treatment. Recruitment will occur primarily through community-based organizations and cancer registries. Participants will be randomly assigned either to a control condition to write about neutral topics or to one of two intervention conditions, self-regulation or self-cultivation, both of which aim to promote adaptive cognitive processes but differ in how they achieve this goal. The self-regulation intervention culturally adapts a Western expressive writing paradigm and incorporates emotional disclosure, whereas the self-cultivation intervention originates from Asian cultural values without disclosing emotions. Participants in all three conditions will be asked to write in their preferred language for three 30-minute sessions. The primary outcome will be quality of life (QOL) at the 6- and 12-month follow-ups, and the secondary outcomes will be perceived stress, stress biomarkers, and medical appointments for cancer-related morbidities.

**Discussion:**

This project will be the first large RCT to test culturally based brief interventions to improve QOL and reduce stress among Chinese immigrant BCSs. This project is expected to address two important needs of Chinese immigrant BCSs: their unmet psychological needs and the lack of culturally competent mental health care for Chinese immigrant BCSs. The immediate product of this line of research will be empirically evaluated, culturally responsive interventions ready for dissemination to Chinese immigrant BCSs across the United States.

**ClinicalTrials.gov identifier:**

NCT04754412.

## Introduction

Cancer, a leading cause of death in the US, affects 38.4% of men and women during their lifetime [1]. In 2022, there were an estimated 18.1 million cancer survivors in the US [2] with $208.9 billion spent on cancer care in 2020 [3]. While curative cancer treatment is a top priority, quality of life (QOL) is also recognized as a key indicator in cancer clinical trials, encompassing physical, functional, social, and emotional domains [4]. Poor QOL values in cancer patients may indicate shorter survival [5, 6], increased healthcare resource utilization [7], and higher healthcare costs [8]. Therefore, as advancements in cancer treatment extend survival, improving long-term QOL of cancer survivors is crucial.

While survival rates for breast cancer, the most common cancer in women, have been increasing, the diagnosis and treatment process can be stressful, contributing to cancer progression and mortality [9]. Breast cancer survivors (BCSs) often face emotional, social, and physical challenges post-treatment [10–13]. The Institute of Medicine recommends integrating psychosocial care into cancer treatment [14] to enhance psychological functioning, alleviate stress-related biological responses, and potentially extend survival [15, 16]. However, such psychosocial interventions are rarely accessible to minority BCSs, especially immigrant survivors who encounter challenges due to limited English proficiency, low health literacy, and/or a lack of culturally competent health care. Therefore, immigrant BCSs face decreased QOL [17, 18] and increased stress [19] and cancer burden compared to their White counterparts.

Although breast cancer shows the fastest-growing incidence among Asian American women [20, 21], little attention is given to this population. Approximately 63% of Chinese Americans, the largest subgroup of Asian Americans (24%) [22], are immigrants [23]. Chinese immigrant BCSs exhibit higher mortality rates than US-born Chinese American BCSs [24] and report poorer QOL than non-Hispanic White BCSs [25]. They also experience significant cancer-related stress and unmet psychosocial needs, including isolation, loneliness, shame associated with cancer [26–31], and fear and anxiety about recurrence, death, pain, and suffering [27, 31, 32]. In addition, there is a shortage of mental health professionals specializing in the psychosocial care of Chinese American BCSs, and few have the necessary language skills to assist those with limited English fluency [33]. The scarcity of culturally competent mental healthcare increases health disparities and unmet psychological and physical health needs in this population.

Expressive writing interventions, as demonstrated in previous studies [34–36], increase the physical and psychological well-being of diverse populations. It allows individuals, especially immigrants, to express their thoughts and emotions freely in any written language, breaking down language barriers and addressing cancer-related stigma within minority communities [37]. Previous research primarily focused on the health benefits of expressive writing for non-Hispanic White BCSs, emphasizing emotional disclosure in writing instructions. Based on the recommendations by the National Institutes of Health Expert Panel to culturally adapt and empirically test interventions validated among White individuals for minority populations [38], our pilot study [37] tailored expressive writing interventions for Chinese immigrant BCSs. Results showed substantial improvements in participants’ QOL and reductions in posttraumatic stress, with the interventions well-accepted by Chinese immigrant BCSs. Another preliminary study revealed that Chinese immigrant BCSs who wrote about stress first, followed by emotions and benefit finding (i.e., writing about positive thoughts and feelings regarding their experience with breast cancer) experienced the greatest improvement in QOL.

Building upon these findings, this study will be the first large randomized controlled trial (RCT) aimed at testing the health benefits of expressive writing interventions on improving QOL and reducing stress among Chinese immigrant BCSs (Aim 1). We hypothesize that interventions will improve QOL, reduce perceived stress and medical appointments for cancer-related morbidities, and normalize levels of stress biomarkers. The primary outcome is QOL, and the secondary outcomes are perceived stress, stress biomarkers, and medical appointments for cancer-related morbidities. Secondary objectives include characterizing how acculturation moderates the effects of expressive writing interventions (Aim 2) and identifying mechanisms explaining their benefits (Aim 3). We hypothesize that individuals highly acculturated to the dominant American culture will benefit more from the self-regulation intervention, while those deeply enculturated toward the heritage Asian culture will benefit more from the self-cultivation intervention. We also expect that expressive writing will improve posttraumatic growth, enhance relationship harmony, and reduce self-stigma, ultimately contributing to an improved QOL.

## Material and methods

### Study design

**Fig 1:**
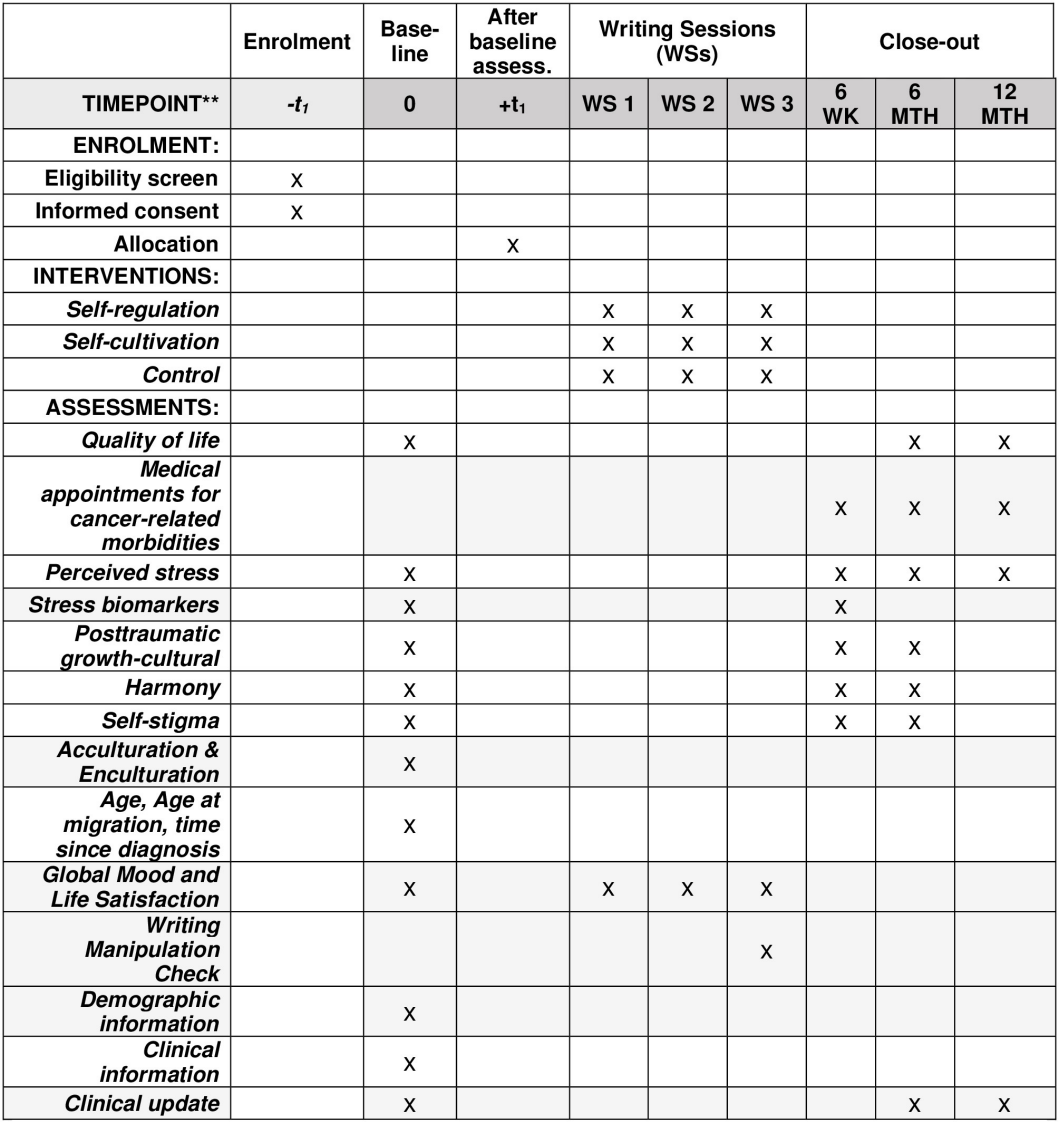
Schedule of enrollment, interventions, and assessments.

We will conduct a randomized controlled trial among Chinese immigrant BCSs who were diagnosed with stage 0-III breast cancer and have completed primary treatment. Participants will be randomly assigned to two intervention groups or the control group. Health outcomes will be assessed at baseline, 6 weeks, and 6 and 12 months after the baseline (Fig 1). All study materials delivered to participants will be in Chinese or English, depending on the participants’ preference.

### Participants

We will recruit 240 Chinese immigrant BCSs diagnosed with stage 0–III breast cancer and within 5 years of completion of primary treatment. The duration of an individual’s participation in the study is 12 months. It is anticipated that enrollment will last 4 years, and the study will be completed in 5 years. Participants will receive up to $200 as compensation for finishing the study and prorated compensation if they withdraw.

### Eligibility criteria

The inclusion criteria are: 1) Being diagnosed with stage 0–III breast cancer; 2) Having completed primary treatment, including surgery, chemotherapy, radiation therapy, immunotherapy, and/or targeted therapy within the preceding 5 years; 3) Foreign-born Chinese women (aged 18 and older) who lived in the US for at least 6 months in the past.

### Recruitment

Participants will be recruited primarily through three community partners: 1) Herald Cancer Association (HCA) and its local offices in Southern California and New York, 2) Shanti Project organization in San Francisco, and 3) the Light and Salt Association in Houston. Through community outreach, these organizations are well connected with local Chinese immigrant BCSs.

Advertising in social media in the Chinese community will be particularly helpful to recruit isolated Chinese immigrant BCSs who are not connected to community-based organizations. IRB-approved advertisements will be placed in popular Chinese media and where Chinese immigrants congregate (e.g., community centers, churches, cultural events, and popular supermarkets).

Patients eligible for inclusion will also be identified through MD Anderson patient databases. Texas and California cancer registries will be utilized if needed.

### Power analysis

Power analysis was performed mainly for the comparison between each intervention group and control group of each primary endpoint (QOL evaluated at 6 or 12 months) using nQuery Advisor Version 7.0 (Statistical Solutions Ltd). A sample size of 64 in each group (80 original, 20% attrition at 12 months) will have at least 80% power to detect a difference in mean QOL of 0.60 standard deviation (SD) using a two-sample *t-*test with a two-sided 0.0125 significance level (with a Bonferroni adjustment for the four primary comparisons). The t-test is appropriate to estimate power because the primary hypothesis is specified as contrasts at specific time points. The effect size of 0.60 was estimated from a preliminary study [39], which compared expressive writing with the control at 6 months. For the same reason, we expect that the self-cultivation intervention condition will have an effect size of 0.6 at the follow-ups. The same sample size will have 80% power to detect a standardized effect size of 0.499 (a medium effect size) in between-group differences in cortisol slopes and perceived stress, to detect a standardized effect size of 0.251 of the interaction between acculturation and treatment conditions (nQuery 7.0), and to detect a mediation effect (standardized coefficient of 0.35 in the b path and standardized between-group difference of 0.60) [40], with a 0.05 two-sided significance level.

### Randomization and blinding

Participants will be randomized to three conditions (control, self-regulation, and self-cultivation) by using a form of covariate-adaptive randomization called minimization, in which participant characteristics are used to assign them to intervention conditions [41, 42]. We will randomize participants based on three variables: time since diagnosis (<12 months, 12 months to 3 years, >3 years to 5 years), disease stage (0, I, II, or III), and age of migration (<18 years or ≥18 years). The procedure results in overall group balance in those three variables [43]. Randomization will be performed by using the Clinical Trial Conduct website developed by the Department of Biostatistics at The University of Texas MD Anderson Cancer Center [44].

To minimize bias, 1) researchers who analyze outcomes will be blinded to participants’ assignments, and 2) participants will be told that the study aims to understand their experience as BCSs through longitudinal surveys and written essays. Participants will not be told that there will be “control” and “intervention” conditions, and a neutral study title (*“My Journey” Study*) will be used on participant study materials.

### Intervention

Participants will be randomly assigned either to a control condition to write about neutral topics or to one of two intervention conditions, self-regulation or self-cultivation. Both conditions aim to promote adaptive cognitive processes but differ in how they achieve this goal. The self-regulation intervention culturally adapts a Western expressive writing paradigm and incorporates emotional disclosure, whereas the self-cultivation intervention originates from Asian cultural values and does not include the disclosure of emotions. Following the baseline assessment, participants in all three conditions will be asked to write in their preferred language in three 30-minute sessions. Note that this is a guideline, and participants are considered adherent to instructions if they write for 15 minutes or more. The specified duration aligns with the common range of 15–30 minutes observed in expressive writing paradigms reported to date [34–36].

Intervention materials will be delivered to participants electronically (email) or through mail, depending on participants’ preference. There will be an option to switch between the online and mail submission methods. All participants will receive reminders through email or text throughout the intervention.

Participants in the control condition will be asked to write about factual aspects of their cancer diagnosis and treatment in the first two sessions and about their diet, exercise, and sleep habits in the third session. Participants in the self-regulation condition will be asked to write about the stress associated with cancer and coping strategies in session 1, deepest feelings about the breast cancer experience in session 2, and positive thoughts and feelings (benefit finding) about their breast cancer journey in session 3. Participants in the self-cultivation condition will be asked to write about their strengthened relationships with others in session 1, personal growth in session 2, and gratitude towards others or reflections on how their stories may benefit others in session 3, based on their experiences with cancer. Following established procedures [45], participants will submit completed essays to our research team for qualitative data analysis.

#### Intervention fidelity

We will check fidelity in several independent ways to increase confidence: 1) Two independent raters, blinded to condition assignment, will read the essays and infer condition assignment. The percentage of match between rated and actual condition assignment will be computed. 2) After the writing sessions, participants will be asked the extent to which their essays are “meaningful” and “reveal emotions” using a 7-point scale (0 = not at all, 6 = a lot) [46]. We expect a higher reported extent of essays revealing negative emotions in the self-regulation intervention compared to the control in sessions 1 and 2. Additionally, we expect that the reported meaningfulness of essays will surpass that of the control in all sessions for each intervention. 3) Following standard procedures [45], we will analyze the written essays using Linguistic Inquiry and Word Count (LIWC) software [47]. We anticipate that self-regulation writing will contain more emotional words in sessions 1 and 2, and more cognitive words in session 3 than the control writing, whereas self-cultivation writing will include more cognitive words than the control writing in all three sessions.

### Procedures involved

At study baseline, each participant will receive a welcome package, which includes a welcome letter and a checklist of tasks to complete. Saliva collection kits and saliva questionnaires for both baseline and 6-week follow-up will be mailed to participants. Following the baseline assessments, participants will complete the writing tasks through their preferred method—either pen and paper or on-line through Research Electronic Data Capture (REDCap) [48]. Questionnaires will be distributed to participants via mail or email at baseline and 6-week, 6-month, and 12-month follow-ups. All study materials provided to participants will be available in Chinese or English, based on their preference. These mailing procedures for data collection have been effective in our preliminary studies, achieving response rates of over 90% among participants who completed expressive writing interventions [39, 45].

#### Assessments

Table 1 shows the assessment schedule. The outcomes including quality of life and stress will be self-reported at baseline and the 6- and 12-month follow-ups. We will assess stress biomarkers at baseline and 6-week follow-up [49]. We will also assess self-reported perceived stress at the 6-week follow-up so that objective and subjective measures of stress are collected at the same time. Following established procedures [50], we will ask participants to prospectively record cancer-related medical visits and reasons for the visits 1 month before the 6 week, 6- and 12-month follow-ups.

**Table 1:** Constructs, measures, and assessment schedule.

Role		Construct	Measures	Base-line	6 WK	6 MTH	12 MTH
**Primary outcome**		Quality of life	Functional Assessment of Cancer Therapy-Breast (FACT-B)	x		x	x
**Secondary outcomes**		Medical appointments for cancer-related morbidities	Prospectively recorded medical appointments for non-routine cancer-related problems (e.g., breast symptoms)		x	x	x
		Perceived stress	Perceived Stress Scale (PSS)	x	x	x	x
		Stress biomarkers	Saliva samples	x	x		
**Mediators**		Posttraumatic growth-cultural	Posttraumatic Growth Inventory (PTGI)—Cultural	x	x	x	
		Harmony	Harmony Scale (self-developed)	x	x	x	
		Self-stigma	Self-Stigma Scale-Short Form	x	x	x	
**Moderators**	**Hypothesized**	Acculturation & Enculturation	Stephenson Multigroup Acculturation Scale	x			
	**Exploratory/Potential**	Age, Age at migration, time since diagnosis	Demographic questionnaire Clinical information questionnaire	x			
**Mood Check**		Global Mood and Life Satisfaction	Global Mood and Life Satisfaction Sliders	x	x		
**Writing Manipulation Check**		Writing Manipulation Check	Writing Manipulation Check		x		
**Covariates**		Demographic information	Race/ethnicity, age, education, income, occupation	x			
		Clinical information	Age at diagnosis, time at diagnosis, cancer stage, treatment	x			
		Clinical update	Cancer recurrence	x		x	x

Three potential psychosocial mediators will be self-reported at baseline and at the 6-week and 6-month follow-ups. The assessment of these mediators will allow for inference of mechanisms in longitudinal models. Moderators, clinical information and demographics, will be self-reported at baseline. Clinical information will also be obtained through medical records with patients’ consent. The Global Mood and Life Satisfaction Sliders will be used at baseline and after each writing session to measure mood. Writing Manipulation Check will be used after completing three writing sessions. Nearly all the standardized questionnaires used in the study have been validated in Chinese samples with good internal reliability.

### Outcome variables

#### Primary outcome

Quality of life. The Functional Assessment of Cancer Therapy-Breast (FACT-B) will be used to measure multidimensional QOL (physical, social, emotional, functional, and breast cancer specific). The FACT-B is widely used in the U.S. [51] and validated in Chinese samples [52] with satisfactory internal reliability (.90) in Chinese immigrant cancer survivors [53]. The primary outcome, QOL, will be indicated by the total score of the four dimensions (physical, social, emotional, and functional). This will allow for a comparison with other studies that involve cancer survivors.

#### Secondary outcomes

Medical appointments for cancer-related morbidities will include appointments for non-routine cancer-related problems (e.g., lymphedema, breast symptoms, or possible recurrence) but exclude scheduled check-ups and non-routine medical appointments for other problems, such as flu symptoms. A previous study found that the rate of agreement of patients’ reports and medical records was 92%, supporting the accuracy of patient reporting [50]. These medical appointments will be coded as a function of reason for the visit (i.e., routine and non-routine cancer-related and non-cancer-related appointments) by raters unaware of participants’ conditional assignment. Non-routine cancer-related visits will be verified through medical records and counted as medical appointments for cancer-related morbidities.

Perceived stress. The Perceived Stress Scale [54] is a psychological instrument widely used for measuring the perception of stress. Its four items measure the degree to which situations in one’s life are appraised as stressful, with good internal reliabilities (0.76–0.80) among Chinese immigrant cancer survivors.

Biomarkers. Following the recommended procedure of assessing stress biomarkers in RCTs, stress-related biomarkers (salivary cortisol and alpha-amylase) will be assessed via saliva samples collected four times per day over two consecutive days before and after the intervention [49]. Salivary cortisol and alpha-amylase levels will be assessed at baseline and at the 6-week follow-up, four times a day. Sampling will occur 20 minutes after waking in the morning, at 12 pm, 5 pm, and 9 pm, over two consecutive days [55]. Following established procedures [55, 56], participants will receive detailed instructions for self-collecting their saliva. Patients will be instructed to refrain from eating, chewing gum, exercising, smoking, brushing teeth, and drinking anything except water for one hour before sampling. Additionally, they will be asked to provide information on comorbid conditions (e.g., autoimmune disorders) and medications that could affect cortisol or alpha-amylase levels (e.g., prednisone, dexamethasone, and other steroids) during the sampling period. They will be given a medical bottle equipped with a Medication Event Monitoring Systems Cap (AARDEX Group, Belgium [57]), an electronic device that has been shown to reliably track the time a saliva sample is taken [58, 59]. Patients will be asked to mail their samples to our laboratory soon after collection. Returning the sample within 5 days is a general guideline based on the feasibility and sample quality. However, it is acceptable for participants to mail their saliva samples within 2–4 weeks because salivary cortisol is stable at room temperature for 2–4 weeks [56] and alpha-amylase has been shown to be stable under a range of conditions [60]. These procedures for saliva sample collection are routinely used in the field [56]. The samples will be stored in our laboratory at -20°C until they are delivered in a large number to be analyzed at a well-established independent laboratory. Research personnel will be responsible for the transmission of saliva samples.

Exploratory secondary outcomes of the study include fear of cancer recurrence, spirituality, sleep, fatigue, and breast cancer specific concerns (the FACT-B breast dimension).

#### Moderating variables

Acculturation and enculturation will be measured using the Stephenson Multigroup Acculturation Scale [61]. Acculturation toward the dominant American culture will be measured using the Dominant Society Immersion subscale, which has 5 items and good internal reliability (α = 0.86) [62, 63], and enculturation toward the heritage culture will be measured using the Ethnic Society Immersion Subscale, which has 5 items and good internal reliability (*α =* 0.90) [62, 64]. Furthermore, potential moderators such as time of diagnosis, age, and age of migration will be self-reported at baseline.

Additional exploratory moderating variables will be measured using standardized scales:

- Avoidance will be measured using avoidance subscale of the Impact of Event Scale (IES) [65], which has good internal reliability (α = 0.77).
- Maintain Harmony will be measured using the maintain harmony subscale of the Brief Collectivism Questionnaire [66], which was developed to assess general collectivism in Asian culture, while capturing its diverse attitudinal and behavioral manifestations. It has 3 items and good internal reliability (α = 0.73).
- Collectivism and emotional control will be measured using the collectivism (3 items) and emotional control (3 items) subscales of the Asian American Values scale, which has good internal reliabilities (α = 0.82–0.92 for collectivism subscale and α = 0.79–0.84) [67].
- Ambivalence over emotional expression will be measured using the Ambivalence over Emotional Expressivity Questionnaire, which has 5 items and good reliability (α = 0.80) [68].

#### Mediating variables

Posttraumatic growth will be measured using the Posttraumatic Growth Inventory [69], which assesses perceived positive changes as a result of a specific traumatic event and has good internal reliability (*α =* 0.93) [69] and validity among Chinese cancer survivors [70].

Self-stigma will be measured using a modified version of the 4-item Self-Stigma Scale-Short Form [71] with good reliability (*α*≥0.90). It assesses the internalized prejudices and negative feelings individuals have toward themselves due to having breast cancer.

Harmony will be measured using a self-developed harmony scale (S1 Text), which assesses participants’ perceived agreement of ideas, feelings, and actions with others. It consists of 4 subscales: affective (3 items), behavioral (2 items), cognitive (2 items), and interpersonal relationships (5 items).

Additional exploratory mediating variables will be measured using standardized scales:

- Gratitude will be measured using Gratitude Questionnaire–Six Item Form (GQ-6) [72], which has six items that measure span, frequency, intensity and density of gratitude and primarily focuses on the unidimensional emotional component of gratitude. It has good internal reliability (α = 0.87).
- Cognitive Appraisal scale will be used to measure how participants appraise the highly stressful events they have experienced, which has 13 items and good internal reliability (α = 0.92) [73].
- Self-Compassion will be measured using the self-kindness subscale (5 items) and the mindfulness subscale (2 items) of the Self-Compassion Scale, which has good internal reliability (α = 0.86) [74].
- Loneliness will be measured using the 3-item version of University of California, Los Angeles (UCLA)-Loneliness Scale, which has good internal reliability (α = 0.88) [75].
- Cancer-related identity will be measured using a question, “When you think about yourself in relation to your cancer, how much does each of these phrases describe you?” (1) a victim of cancer, (2) a cancer patient, (3) a person who has had cancer, (4) a survivor, and (5) a person who recovered, each rated from 1 (not at all) to 5 (Very much) [76].
- Intrusive Thoughts will be measured using the intrusion subscale of the Impact of Event Scale (IES)-6, which has 2 items and good internal reliability (α = 0.80) [77].

#### Mood check

Mood will be measured using the Global Mood and Life Satisfaction Sliders [78]. Participants will be asked to rate their general affect (“How do you feel right now?”) and life satisfaction (How satisfied with your life are you right now?”). The scores range from 0 to 100 with a higher score indicating greater satisfaction.

#### Writing manipulation check

Writing manipulation will be measured using four questions, “Overall, how personal were the essays that you wrote?”, “Overall, how much did you reveal your emotions in what you wrote?”, “Other than receiving monetary compensation, to what degree has this study been valuable or meaningful for you?”, and “During the writing session, did you gain any new perspective of the disease.” The scores range from 0 (not personal/not at all) to 6 (personal/a lot).

### Data analysis

Before we begin inferential procedures, we will conduct extensive descriptive analyses on the data collected. Distributional assumptions will be evaluated, and if indicated, normalizing (e.g., log) transformations or robust procedures will be used. Appropriate procedures to assess the validity and reliability of questionnaires will be used.

Given the longitudinal nature of the study, we will use linear mixed-effects models (LMMs) to assess the effects of the interventions on outcomes. Treatment condition and time will be included and tested in the model, as well as their interaction, with adjustment for covariates [79] including the baseline outcome value, time since diagnosis, and disease stage. If needed, we will also control for potential confounders such as demographic or other clinical variables correlated with outcome variables. Potential heterogeneity across recruitment sites will be accounted for by adding site-specific random intercepts. The repeated-measures correlation structure will be selected based on the Bayesian information criterion (BIC). The primary outcome will be a composite scale of QOL based on the FACT-B total score. QOL at 6- and 12-month follow-ups for both intervention conditions will be tested against the control condition within the LMMs based on appropriately constructed contrasts, each with a two-sided 0.0125 level of significance, to ensure that the overall type I error rate across the four tests does not exceed 0.05. Controlling the overall type I error rate for testing the four primary hypotheses will help increase the reproducibility of our main study findings.

All other analyses described below will use a two-sided significance level of 0.05 without adjustment for multiple testing. The corresponding results will thus be interpreted as hypothesis-generating. The above LMMs will be repeated for medical appointments and perceived stress as outcomes. We will conduct linear regression analyses with stress biomarkers (cortisol and alpha amylase) as the outcomes. The average levels and average slope of change over 2 days at the 6-week follow-up will be used as the outcome of interest for each stress biomarker [80, 81]. We will control potential confounding variables such as medical conditions and medications.

To characterize the moderation effect of acculturation/enculturation on the intervention, we will use LMMs like those above but test additional interaction effects between acculturation/enculturation subscale composite scores and intervention conditions. Items to assess the language aspects in the acculturation/enculturation subscales will also be explored as potential moderators. Furthermore, we will test interaction effects between intervention conditions and additional potential moderators (time since diagnosis, age, age of migration, intervention delivery modes [receiving instructions via mail and writing by hand vs. receiving instructions and writing online]) because the effects of the interventions may also depend on these factors.

To identify potential mechanisms by which the expressive writing intervention yields health benefits, we will conduct a longitudinal mediation analysis [82]. Specifically, we will use measures of QOL outcomes at 6- and 12-month follow-ups and each potential mediator (posttraumatic growth, relationship harmony, and self-stigma) at 6-week and 6-month follow-ups, respectively, in the a and/or b paths of the mediation models (each path being a LMM [82]). We will use bootstrapping in testing for the indirect effect of each mediator on the QOL outcome [83]. We will also explore multiple mediator models in case more than one mediator emerges [84].

#### Missing data and dropouts

The LMMs, as a likelihood-based regression method, will produce valid (e.g., asymptotically unbiased) estimates of effects provided that the missingness probability depends only on the observed variables in the model (missing at random). However, data may not be missing at random; thus, we will conduct sensitivity analyses assuming different missing data mechanisms, especially when the attrition rate is moderate or high (e.g., >10%) and/or the attrition is unbalanced across intervention conditions. We will consider multiple imputation approaches based on relevant baseline participant characteristics to account for the missing-at-random mechanisms. We will also explore pattern-mixture and selection models to account for potential missing-not-at-random mechanisms [85].

### Data management plans

Quality assurance procedures will be developed for data management. Study investigators and staff will maintain quality assurance procedures for all data collected. Measures to ensure quality control of collected data include pre-entry review by data management staff, review of entered data against scanned paper copies, frequent data audits that involve randomly selecting a sample of data and comparing it against the entered data, using aggregate reports to spot errors, and developing a data dictionary to systematically catalog and communicate the structure and content of the data.

All paper questionnaire packets and essays will be stored in a secure and locked location. All protected health information (PHI) records obtained will be kept in accordance with relevant HIPAA requirements. Participants’ quantitative assessments, essays, and saliva samples will be coded only by participant ID numbers. All PHI will be removed from the data when it is exported for analysis. Data will be cross-checked to ensure accuracy.

### Ethical considerations and declarations

This study has been approved by the Institutional Review Board of the University of Texas MD Anderson Cancer Center. Written informed consent from all participants will be obtained for this study. Consent documents will be in Chinese with English documents provided upon request. All research personnel have completed all required human subjects research training. This trial is registered at ClinicalTrials.gov (ID NCT04754412, last update: November 01, 2023).

## Discussion

Expressive writing interventions have been shown to benefit BCSs in previous studies, but most studies focused on non-Hispanic White BCSs. This study will be the first large RCT to test culturally originated brief expressive writing interventions to improve QOL and reduce stress among Chinese immigrant BCSs. Moreover, in most previous studies, writing instructions primarily involved disclosing one’s deepest thoughts and feelings. However, less acculturated Asian Americans may benefit from writing instructions facilitating more cognitive than emotional processes [86].

This study is expected to address two important unmet needs of Chinese immigrant BCSs: psychological needs and culturally competent mental healthcare. To this end, we will evaluate two culturally adapted interventions, differing in their inclusion of a Western component previously deemed essential. The self-regulation intervention culturally adapts a Western expressive writing paradigm and incorporates emotional disclosure, whereas the self-cultivation intervention originates from Asian cultural values and does not include emotional disclosure. Furthermore, we will characterize how acculturation moderates the effects of expressive writing interventions. We hypothesize that those highly acculturated to the dominant American culture will benefit more from the self-regulation intervention and those highly enculturated toward the heritage Asian culture will benefit more from the self-cultivation intervention. The proposed interventions have the potential to enhance participants’ mental and physical health by improving QOL, reducing perceived stress, decreasing medical appointments for cancer-related morbidities at 6- and 12-month follow-ups, and normalizing stress biomarker levels at the 6-week follow-up.

The design of this study has some limitations. The study only includes Chinese immigrant BCSs who have completed treatment, limiting the generalizability of the results. Expressive writing is highly scalable, as it requires minimal personnel and equipment. Therefore, this empirically evaluated, culturally responsive intervention has the potential to be adapted for other Asian American cancer survivors across the United States, but further studies are warranted to explore its applicability and efficacy in these populations. Because we are testing acculturation and incorporating cultural values in the intervention, the intervention may not be directly applicable to cancer survivors of other racial or ethnic minority groups (Blacks). Similar interventions can be developed considering the cultural values of the population of interest, but those interventions should be empirically tested before implementation in the community.

## Supporting information

S1 Text Harmony scale. (PDF)

S1 Protocol Writing to heal: A culturally based brief expressive writing intervention for Chinese immigrant breast cancer survivors. (PDF)

S1 Checklist SPIRIT outcomes 2022 checklist. (PDF)
