# Supplementary material for: Study protocol for writing to heal: A culturally based brief expressive writing intervention for Chinese immigrant breast cancer survivors
Source: PLoS One. 2024 Sep 26;19(9):e0309138. doi: 10.1371/journal.pone.0309138 (PMC11426517; doi:10.1371/journal.pone.0309138)
Supplement: S1 Text — (PDF) [file pone.0309138.s001.pdf]

Harmony Scale

A. Think about the most important people in your daily life. Please rate your level of agreement with the following statements to indicate how you perceive your relationships with them **<u>over the past MONTH</u>**.

	**Strongly disagree**	**Disagree**	**Neutral**	**Agree**	**Strongly agree**
1. I have felt peace in my relationships.	1	2	3	4	5
2. There is a feeling of togetherness with my family members.	1	2	3	4	5
3. I do not feel conflict with those important to me.	1	2	3	4	5
4. My family members and I have gotten along well with each other.	1	2	3	4	5
5. I and those important to me support each other by compromising when problems come up.	1	2	3	4	5
6. Even when I have disagreed with my family members, I have tried to tolerate their opinions.	1	2	3	4	5
7. I and those important to me believe we agree with each other on our ideas (even if we initially have differences).	1	2	3	4	5

Please think of five most important relationships to you using the list below. Next, consider the degree of harmony that you believe has been reached in each of these 5 relationships. Use the following 7 points Likert-scale to assess each relationship and write down the appropriate scores in the space provided below.

**Spouse**	**Father**	**Mother**	**Son**	**Daughter**	**Brother**
**Sister**	**Father-in-law**	**Mother-in-law**	**Son-in-law**	**Daughter-in-law**	**Grandson**
**Granddaughter**	**Friend**	**Neighbor**	**Colleague**	**Other（Please specify____）**	

**Relationship**	**The degree of harmony in your relationship with the other person**						
	**Not At all**	**A very small degree**	**A small degree**	**Neutral**	**A moderate degree**	**A great degree**	**A very great degree**
1. ______________________________	1	2	3	4	5	6	7
2. ______________________________	1	2	3	4	5	6	7
3. ______________________________	1	2	3	4	5	6	7
4. ______________________________	1	2	3	4	5	6	7
5. ______________________________	1	2	3	4	5	6	7
