# Supplementary material for: Study protocol for writing to heal: A culturally based brief expressive writing intervention for Chinese immigrant breast cancer survivors
Source: PLoS One. 2024 Sep 26;19(9):e0309138. doi: 10.1371/journal.pone.0309138 (PMC11426517; doi:10.1371/journal.pone.0309138)
Supplement: S1 Protocol — (PDF) [file pone.0309138.s002.pdf]

2020-0708: Writing to Heal: A Culturally Based Brief Expressive Writing Intervention for Chinese Immigrant Breast Cancer Survivors

PROTOCOL TITLE: Writing to Heal: A Culturally Based Brief Expressive Writing Intervention for Chinese Immigrant Breast Cancer Survivors

PRINCIPAL INVESTIGATOR:

Qian Lu, M.D., Ph.D.
Department of Health Disparities Research
The University of Texas MD Anderson Cancer Center
Phone: 713-745-8324
Email: qlu@mdanderson.org

2020-0708: Writing to Heal: A Culturally Based Brief Expressive Writing Intervention for Chinese Immigrant Breast Cancer Survivors

Table of Contents

1.0 Study Summary 3
2.0 Objectives 4
3.0 Background 4
4.0 Study Endpoints 6
5.0 Study Intervention/Investigational Agent 6
6.0 Procedures Involved .... **Error! Bookmark not defined.**
7.0 Data and Specimen Banking 13
8.0 Sharing of Results with Subjects 15
9.0 Study Timelines* 15
10.0 Inclusion and Exclusion Criteria* 15
11.0 Vulnerable Populations 16
12.0 Local Number of Subjects 16
13.0 Recruitment Methods 16
14.0 Withdrawal of Subjects 16
15.0 Risks to Subjects 16
16.0 Potential Benefits to Subjects 16
17.0 Data Management and Confidentiality 17
18.0 Provisions to Monitor the Data to Ensure the Safety of Subjects 17
19.0 Provisions to Protect the Privacy Interests of Subjects 17
20.0 Compensation for Research-Related Injury 17
21.0 Economic Burden to Subjects 17
22.0 Consent Process 17
23.0 Setting 18
24.0 Resources Available 19

1.0 Study Summary

**Study Title**	Writing to Heal: A Culturally Based Brief Expressive Writing Intervention for Chinese Immigrant Breast Cancer Survivors
**Study Design**	Randomized Controlled Trial
**Primary Objective**	To use a randomized controlled trial (RCT) to test the health benefits of expressive writing that is culturally adapted for Chinese immigrant breast cancer survivors (BCSs).
**Secondary Objective(s)**	**1.** To determine the health benefits of expressive writing compared with the control condition.<br>**2:** To characterize how acculturation moderates the effects of expressive writing interventions.<br>**3:** To identify mechanisms explaining the benefits of the interventions using mixed methods.
**Research Intervention(s)/ Investigational Agent(s)**	Psychosocial – Expressive writing
**IND/IDE #**	N/A
**Study Population**	Adult Chinese American breast cancer survivors
**Sample Size**	240
**Study Duration for individual participants**	12 months

2.0 Objectives

The study uses a randomized controlled trial (RCT) to test the health benefits of expressive writing that is culturally adapted for Chinese immigrant breast cancer survivors (BCSs). We will recruit Chinese immigrant BCSs (N=240) diagnosed with stage 0-III breast cancer and within 5 years of completion of primary treatment. Recruitment will occur primarily through community-based organizations and from The University of Texas MD Anderson Cancer Center patient database. Participants will be randomly assigned either to a control condition to write about neutral topics or to one of two intervention conditions, self-regulation or self-cultivation, which both aim to promote adaptive cognitive processes but differ in how they achieve this goal. The self-regulation intervention incorporates a traditional Western expressive writing paradigm, whereas the self-cultivation intervention incorporates Asian cultural values. Participants in all three conditions will be asked to write in their preferred language during three 30-minute sessions.

The primary outcome will be quality of life (QOL) at the 6 and 12 month follow-ups and the secondary outcomes will be perceived stress, stress biomarkers, and medical appointments for cancer-related morbidities.

Aim 1: To determine the health benefits of expressive writing compared with the control condition.
Hypothesis 1.1: Both interventions will improve QOL and reduce perceived stress and medical appointments for cancer-related morbidities at the 6- and 12-month follow-ups.
Hypothesis 1.2: Both interventions will reduce perceived stress and normalize levels of stress biomarkers at the 6-week follow-up.

Aim 2: To characterize how acculturation moderates the effects of expressive writing interventions.
Hypothesis 2.1: Those highly acculturated to the dominant American culture will benefit more from the self-regulation intervention and those highly enculturated toward the heritage Asian culture will benefit more from the self-cultivation intervention.

Aim 3: To identify mechanisms explaining the benefits of the interventions using mixed methods.
Hypothesis 3.1: Expressive writing will improve posttraumatic growth and relationship harmony and reduce self-stigma, which will in turn improve QOL, investigated using quantitative methods.
Research question 3.2: What culturally specific factors explain the benefits of the interventions, investigated using qualitative methods?

3.0 Background

Cancer Survivors Need Psychosocial Care

Cancer is one of the leading causes of death and disease in the United States. Approximately 38.4% of men and women will be diagnosed with cancer at some point in their life.[1] In the United States, there were an estimated 15.5 million cancer survivors in 2016,[1] and $147.3 billion was spent on cancer care in 2017.[2] Although providing curative treatment remains the number one priority, QOL is now considered an important indicator of disease burden[3] and an endpoint in cancer clinical trials. In cancer, QOL has been defined as a person's well-being generally encompassing physical, functional, social, and emotional domains.[4] Poor QOL among cancer patients predicts shorter survival time,[5,6] more subsequent health care resource utilization,[7] and higher subsequent healthcare costs.[8] As new technology in *cancer* treatment is helping patients live longer, maintaining adequate long-term QOL throughout cancer survivorship has become a principal concern.

Breast cancer is the most commonly diagnosed cancer in women. Breast cancer survival rates are increasing; however, breast cancer diagnosis and treatments remain stressful, and the chronic

stress can linger long after treatment ends. Chronic stress accelerates cancer progression and increases mortality.[9] As breast cancer survivors (BCSs) face numerous emotional, social, and physical challenges following breast cancer treatment,[10-13] the Institute of Medicine recommends that cancer care integrate psychosocial care.[14] Psychosocial interventions improve psychological functioning, reduce stress-mediated biological responses, and even prolong survival.[15,16] However, psychosocial interventions are rarely offered to minority cancer survivors.

Immigrant BCSs Experience Significant Disparities in QOL and Psychosocial Care

Compared to their white counterparts, racial minorities, and particularly minority immigrant BCSs, experience poorer QOL,[17,18] higher distress,[19] and more burden from cancer. Immigrant cancer survivors may face multiple challenges in psychosocial care due to limited English proficiency, low health literacy, and/or lack of culturally competent health care.

Asians passed Hispanics as the largest group of new immigrants to the United States in 2008.[20] Cancer is the leading cause of death for Asian Americans,[21] and breast cancer is the cancer with the fastest-increasing incidence among Asian Americans.[21,22] Despite this increase and the growing Asian American population (17.3 million) in the United States,[23] little attention has been focused on Asian American BCSs. Chinese Americans are the largest subgroup of Asian Americans (comprising 24%),[24] and 63% of Chinese Americans are immigrants.[25] Notably, Chinese immigrant BCSs have a **higher mortality rate** than U.S.-born Chinese American BCSs[26] and report **poorer QOL** and higher self-stigma than non-Hispanic white BCSs.[27] Chinese immigrant BCSs experience significant cancer-related stress and unmet psychosocial needs, including isolation, loneliness, shame associated with cancer,[28-33] and fear and anxiety regarding recurrence, death, pain, and suffering.[29,33,34]

Compounding this problem, few mental health professionals specialize in the psychosocial care of Chinese American BCSs, and even fewer have the language capacity to serve the more than 46% of Chinese Americans with limited English fluency.[35] The lack of culturally competent mental health care for Chinese immigrant BCSs and their unmet psychological and physical health needs represent **significant health disparities experienced by this group**.

Expressive Writing Is a Promising and Highly Disseminable Psychosocial Intervention

Expressive writing is personal and emotional writing without regard to writing conventions such as spelling and punctuation; it enables writers to simply express their thoughts and emotions. Expressive writing may be especially suitable for immigrant BCSs because it overcomes potential linguistic barriers and stigma associated with cancer among minorities.[36] Because it is private, expressive writing allows people to disclose emotions without being stigmatized and without damaging harmony with others. Furthermore, expressive writing interventions can be delivered in any written language to overcome the barrier of limited English proficiency, which is common among immigrant BCSs.

Expressive writing can improve physical and psychological well-being. The expressive writing paradigm was developed by Pennebaker and colleagues, who discovered the health benefits of writing about emotional upheaval.[37] In the typical Pennebaker expressive writing study, participants are assigned randomly to either an intervention group to write about their deepest thoughts and feelings related to a traumatic experience or a control group to write about a neutral topic unrelated to deep feelings, with both groups writing in three 20- or 30-minute sessions.[37] Reviews and a meta-analysis of 146 RCTs of written emotional disclosures revealed that this type of intervention confers a variety of benefits, including increased physical and psychological well-being.[38-40] This paradigm has been adopted by many cancer survivor researchers. Expressive writing has been shown in multiple RCTs to confer health benefits to non-Hispanic white BCSs,[41] including reduced medical appointments for cancer-related morbidities,[42] reduced physical symptoms,[42,43] improved depressive symptoms,[43] and improved perceived social support.[44]

Expressive writing must be culturally adapted. Although the above studies demonstrated that

expressive writing delivered health benefits to BCSs, these studies included mostly non-Hispanic whites, and the writing instruction primarily involved disclosing one's deepest thoughts and feelings (i.e., emotional disclosure). An National Institute of Health Expert Panel has strongly recommended that interventions validated among Caucasians should be culturally adapted for minorities and empirically tested.[45]

Significance of the Expected Research Contribution

This project will be the first large RCT to test culturally adapted brief interventions to improve QOL and reduce stress among Chinese immigrant BCSs. The project is expected to address important unmet needs of Chinese immigrant BCSs and has broad implications for science and practice. This project is expected to address two important unmet needs of Chinese immigrant BCSs: their unmet psychological needs and the lack of culturally competent mental health care for Chinese immigrant BCSs. Expressive writing is highly scalable, as it requires minimal personnel and equipment. The immediate product of this line of research will be empirically evaluated, culturally responsive interventions ready for dissemination to Chinese immigrant BCSs across the United States.

Preliminary Studies

Findings from Dr. Lu's preliminary studies support our hypotheses 1.1, 1.2, 2.1, and 3.1 and form the basis for the design of our proposed interventions.

Study 1. We tested the feasibility of expressive writing among 19 Chinese immigrant BCSs who engaged in three weekly expressive writing tasks and completed 3- and 6-month follow-up assessments. Participants showed improvement in QOL (d=0.62) and posttraumatic stress (d=0.67) at the 6-month follow-up.[36] They highly valued the writing tasks and disclosed thoughts and emotions that they *had not* shared with anyone before, suggesting that expressive writing was well-accepted by Chinese immigrant BCSs, who confided their thoughts in writing.

Study 2. Chinese immigrant BCSs were randomly assigned to a control writing condition or to one of two intervention writing conditions, for which writing instructions differed only in terms of whether emotion or stress was disclosed first.[46] The writing condition in which emotion was disclosed first had only small effects. The writing condition in which participants (N=47) wrote about stress first, then emotion and benefit finding (i.e., writing about positive thoughts and feelings regarding one's experience with breast cancer) produced the greatest increase in QOL from baseline to the 6-month follow-up (d=0.6). We plan to use the same writing instructions in the self-regulation condition in the proposed study.

4.0 Study Endpoints

The primary outcome will be QOL at the 6 month and 12 month follow-ups and the secondary outcomes will be perceived stress, stress biomarkers, and medical appointments for cancer-related morbidities.

5.0 Study Intervention

Design. The study is a randomized controlled trial. Participants will be randomized to three conditions (control, self-regulation, and self-cultivation) by using a form of covariate-adaptive randomization called minimization, in which participants' characteristics are used to assign them to the intervention conditions.[47,48] Minimization results in better marginal group balance in participant characteristics compared with stratification. Minimization provides balanced intervention conditions throughout the randomization process. For the proposed study, which has extended accrual periods, the intervention conditions will remain balanced with respect to participant characteristics that may be related to the time of accrual. We will randomize participants based on three variables: time since diagnosis (<12 months, 12 months to 3 years, >3 years to 5 years), disease stage (0, I, II, or III), and age of migration (<18 years or ≥18 years). The procedure results in overall group

balance in those three variables.[49] The randomization will be performed by using the Clinical Trial Conduct website developed by the Department of Biostatistics at The University of Texas MD Anderson Cancer Center.[50]

Intervention. After the baseline assessment, participants will be asked to write during three sessions. For each session, they are asked to write about 30 minutes. Note that this is a guideline, and participants are considered adherent to instructions if they write for 15 minutes or more. Participants will be asked to report whether they write for at least 15 minutes. This writing-session length is based on the range of 15-30 minutes typical for writing sessions in expressive writing paradigms reported to date, a meta-analysis showing that longer sessions had more benefit,[38-40] and preliminary study 2 showing that writing in 30-minute sessions worked well.

Intervention materials will be delivered to participants electronically (email) or through the mail, depending on participants' preferences. Participants who choose the electronic option will receive emails that contain a personalized link to the writing tasks on REDCap. Participants who choose the mail option will receive the writing instructions by mail. There will be an option to switch between the online and mail submission methods and vice versa. Hardcopies will be provided to participants as a default if REDCap is unavailable. All the participants will receive reminders through email or text throughout the intervention, for example, "this is (researcher's name) from the 'My Journey' Study at The University of Texas MD Anderson Cancer Center. This is a friendly reminder that you have 3 surveys left for the study."

Participants in the control condition will be asked to write about facts regarding their cancer diagnosis and treatment in the first two sessions and about their diet, exercise, and sleep habits in the third session. These writing instructions engaged participants, and the control and intervention instructions yielded similar lengths of essays in preliminary study 2. Participants in the self-regulation condition will be asked to write about stress and coping, emotional disclosure, and benefit finding. During session 1, participants will be asked to write about the stress of having cancer and their strategies for coping with cancer-associated stressors. During session 2, participants will be asked to write about their deepest feelings regarding their breast cancer experience. During session 3, participants will be asked to write about positive thoughts and feelings regarding their breast cancer experience. Participants in the self-cultivation condition will be asked to write about how they have strengthened their relationships with others in session 1, how they have grown personally in session 2, and detailing their gratitude towards others or their experience to help others in session 3. The writing instructions were developed based on preliminary study 3 and the community's input. All writing instructions are in Appendix B. Following established procedures,[51] we will ask participants to return completed essays to our research team. In our pilot studies, participants were comfortable sharing their essays. The essays will be used for qualitative data analysis.

Blinding. To minimize bias, 1) researchers who assess/analyze outcomes will be blinded to participants' assignments (we have successfully achieved such blinding in our preliminary studies), and 2) participants will be told that the study aims to understand their experience as BCSs through longitudinal surveys and written essays. Participants will not be told that there will be "control" and "intervention" conditions and a neutral study title (*"My Journey" Study*) will be used on participant study materials. Participants will be debriefed upon completing the last follow-up and will be asked not to share the information with others to prevent potential contamination until the completion of the study by the research team. Such procedures worked well in our pilot studies and minimized expectation bias among participants (the control group was as engaged as the intervention group, suggested by similar essay lengths between groups). To determine whether expectations differ among groups, expectations of participants will be assessed through both open-ended questions and self-ratings of expected benefits at the end of the third writing session.

Intervention fidelity. We will check fidelity in several independent ways to increase confidence: 1) Following standard procedures,[83] two independent raters, blinded to condition

assignment, will read the written essays and infer condition assignment. The percentage of match between rated and actual condition assignment will be computed. 2) After the writing sessions, participants will be asked the extent to which their essays are “meaningful” and “reveal emotions” using a 7-point scale (0 = not at all, 6 = a lot.)[52] We expect that the reported extent to which essays reveal negative emotions will be greater in the self-regulation intervention than the control in writing sessions 1 and 2 and that the reported meaningfulness of essays will be greater in each intervention than in the control for all sessions. 3) Following standard procedures,[51] we will analyze the written essays using Linguistic Inquiry and Word Count (LIWC) software.[53] We expect that the self-regulation writing will contain more emotional words in writing sessions 1 and 2 and more cognitive words in writing session 3 than the control writing; the self-cultivation writing will contain more cognitive words than the control writing in all three sessions.

6.0 Procedures Involved

Individuals who consent to participate in this study will communicate with community and/or research staff about study procedures. At study baseline, all participants will receive a welcome package including materials such as a welcome letter, gifts, and a checklist of tasks to complete. Participants who elect to complete the study on paper and pen will also receive baseline questionnaires in their welcome package. Saliva collection kits and saliva questionnaires for both the baseline and 6-week follow-up timepoints will be mailed to them. After completing the baseline assessments, participants will complete the writing tasks through their preferred method which can be pen and paper or REDCap. Questionnaire will either be mailed or electronically sent to participants at baseline, and at around 6 week, 6 month, and 12 month of follow-up. All study materials delivered to participants will be in Chinese or English, based on their preference. Participants who choose to complete assessments by hand will receive postage-paid envelopes to mail back their responses. These mailing procedures for data collection worked well in our preliminary studies, with higher than 90% response rates among those who completed expressive writing interventions.[46,51]

All participation will be with fully informed consent, and participants are free to withdraw from the study at any time without penalty. Prior to enrollment, if an otherwise eligible participant reports high levels of distress, such that informed consent and/or completion of the baseline is not possible, the participant will not be enrolled and an appropriate local support resource referral will be offered (see the Referral Letter). The referral will be provided if a participant requests such services or if the staff feels such professional assistance is needed. Referral will also be provided after study enrollment if, at any point, a participant reports very high levels of distress. Participants may be contacted for a number of reasons including but not limited to establishing a connection early on in the study or collecting any missing data, or asking about their experiences and opinions toward the study. Participants will be asked to sign and date source documents they completed.

Assessments

Nearly all of the standardized questionnaires in the study have been validated in Chinese samples with good internal reliability. Questionnaires are provided in Appendix A. Table 1 shows the assessment schedule. Primary outcome, secondary outcomes, mediators, moderators, and clinical information (e.g., medical appointments, cancer recurrence, treatment type, cancer stage, time since diagnosis) will be self-reported at

TABLE 1. Constructs, Measures, and Assessment Schedule

Role	Construct	Measures	Base-line	6 WK	6 MTH	12 MTH
Primary outcome	Quality of life	Functional Assessment of Cancer Therapy-Breast (FACT-B)	x		x	x

Secondary outcome		Medical appointments for cancer-related morbidities[1]	Prospectively recorded medical appointments for non-routine cancer-related problems (e.g., breast symptoms)		x	x	x
		Perceived stress	Perceived Stress Scale (PSS)	x	x	x	x
		Fear of cancer recurrence	FACT21	x	x	x	x
		Spirituality	FACIT-Sp	x	x	x	x
		Sleep	Pittsburgh Sleep Quality Index (PSQI)	x	x	x	x
		Fatigue	Patient Reported Outcomes Measurement (PROMIS Fatigue 4a	x	x	x	x
		Stress biomarkers	Saliva samples	x	x		
Mediators		Posttraumatic growth-cultural	Posttraumatic Growth Inventory (PTGI) - Cultural	x	x	x	
		Harmony	Harmony Scale (self-developed)	x	x	x	
		Gratitude	Gratitude Questionnaire Six Item Form (GQ-6)	x	x	x	
		Cognitive appraisal	Cognitive Appraisal of Health Scale	x	x	x	
		Loneliness (3 item)	UCLA Loneliness Scale (Version 3)	x	x	x	
		Identity	Identity	x	x	x	
		Intrusive thoughts	Intrusive thoughts subscale of the Impact of event scale (IES)	x	x	x	
		Self-stigma	Self-Stigma Scale-Short Form	x	x	x	
		Self-compassion	Self-compassion scale (SCS)	x	x	x	
Moderators	Hypothesized	Acculturation & Enculturation	Stephenson Multigroup Acculturation Scale	x			
	Exploratory/ Potential	Age, Age at migration, time since diagnosis,, delivery mode	Demographic questionnaire Clinical information questionnaire	x			
		Avoidance	Impact of Event Scale (IES) avoidance subscale	x			
		Maintain harmony subscale	Brief Collectivism Questionnaire (BCQ) 3 items in the maintain harmony subscale (see separate sheet)	x			
		collectivism and emotional control subscales	Asian American Values Scale	x			
		Ambivalence over emotional expression	Ambivalence over emotional expression (AEE) 5 items version	x			
Mood Check		Global Mood and Life Satisfaction	Global Mood and Life Satisfaction Sliders	x	x		
Writing Manipulation Check		Writing Manipulation Check	Writing Manipulation Check		x		
Covariates		Demographic information	Race/ethnicity, age, education, income, occupation	x			
		Clinical information	Age at diagnosis, time at diagnosis, cancer stage, treatment	x			
		Clinical update	Cancer recurrence	x		x	x

baseline and 6- and 12-month follow-ups. Clinical information will also be obtained through medical records with patients' consent, a procedure successfully used in pilot studies.

Moderating variables and demographics will be self-reported at baseline. Potential psychosocial mediators will be self-reported at baseline and at the 6-week and 6-month follow-ups. The three assessments of mediators will allow for inference of mechanisms in longitudinal models. We will assess biomarkers at baseline and 6-week follow-up, based on the literature.[54] We will also assess self-reported perceived stress at the 6-week follow-up so that objective and subjective measures of stress are expected to be collected at the same time. The Global Mood and Life Satisfaction Sliders will be used at baseline and after each writing session to measure mood. Writing Manipulation Check will be used after completing three writing sessions.

Outcome variables.

Primary Outcomes:

- Quality of life. The Functional Assessment of Cancer Therapy-Breast (FACT-B) will be used to measure multidimensional QOL (physical, social, emotional, functional, and breast cancer specific). The FACT-B is widely used in the U.S.[55] and validated in Chinese samples,[56] with satisfactory internal reliability (.90) in Chinese immigrant cancer survivors.[57] The primary outcome of quality of life will be indicated by the total score of the four dimensions including physical, social, emotional, and functional. This will allow for comparison with other studies of cancer survivors.

Secondary Outcomes:

- Medical appointments for cancer-related morbidities will include appointments for non-routine cancer-related problems (e.g., lymphedema, breast symptoms, or possible recurrence) but exclude scheduled check-ups and non-routine medical appointments for other problems, such as flu symptoms. Following established procedure,[42] we will ask participants to prospectively record cancer-related medical visits and reasons for the visits 1 month before the 6 week, 6- and 12-month follow-ups. A previous study found that rate of agreement of patients' reports and medical records was 92%, supporting the accuracy of patient reporting.[42] These medical appointments will be coded as a function of reason for the visit (i.e., routine and non-routine cancer-related and non-cancer-related appointments) by raters unaware of participants' conditional assignment. Non-routine cancer-related visits will be verified through medical records and counted as medical appointments for cancer-related morbidities. We have successfully obtained and verified medical records among Chinese BCSs in prior studies.
  Perceived stress. The Perceived Stress Scale[58] is a psychological instrument widely used for measuring the perception of stress. Its 4 items measure the degree to which situations in one's life are appraised as stressful, with good internal reliabilities (0.76~0.80) among Chinese immigrant cancer survivors.

Exploratory outcomes include several psychological health and symptoms.

- Fear of cancer recurrence will be measured by one item, "I worry that my cancer will come back." It has been found to be one of the items best representing the concept of fear of recurrence [59]. On a 5-point Likert scale (0 as not at all, 1 as a little bit, 2 as somewhat, 3 as quite a lot, 4 as very much), a higher score indicates higher fear of recurrence.
- Spirituality: The Functional Assessment of Chronic Illness Therapy-Spiritual Well-Being (FACIT-Sp-12) is a 5-item questionnaire that measures spiritual well-being in people with cancer and other chronic illnesses.[60]

- Sleep: The Pittsburgh Sleep Quality Index (PSQI) is a self-rated questionnaire assessing sleep quality and disturbances over a 1-month time period. Nineteen individual items generate 4 component scores, of which the sum yields a global score.[61]
- Fatigue will be measured using the PROMIS SF v1.0 – Fatigue 4a.[62] Total scores for scale range from 4 to 20, with higher scores indicating greater fatigue. The internal consistency for the PROMIS SF-Fatigue 4a is .90 or higher.[62]
- Biomarkers*.* Following the recommended procedure of assessing stress biomarkers in RCTs, stress-related biomarkers (salivary cortisol and alpha-amylase) will be assessed via saliva samples collected four times per day over two consecutive days before and after the intervention.[54] Salivary cortisol and alpha-amylase levels will be assessed at study entry and at the 6-week follow-up four times a day, starting 20 minutes after waking up in the morning, 1200, 1700, and 2100 hours on each of two consecutive days.[63] Following established procedures,[63,64] patients will be given detailed instructions on collecting their own saliva on their own. Patients will be asked to refrain from eating, chewing gum, exercising, smoking, brushing teeth, and drinking anything except water *for 1 hour before sampling;* and to complete questions (see Appendix A) regarding comorbid conditions (e.g., autoimmune disorders) and medications that may influence cortisol or alpha-amylase levels (e.g., prednisone, dexamethasone, and other steroids) during the sampling period. They will be given a medical bottle equipped with a Medication Event Monitoring Systems Cap (AARDEX Group, Belgium[65]), an electronic device that has been shown to reliably track the time a saliva sample is taken.[66,67] Patients will be asked to mail their samples to our laboratory soon after collection. Retuning the sample within 5 days is a general guideline based on the feasibility and sample quality. However, it is acceptable for the participants to mail the saliva samples within 2 - 4 weeks, because salivary cortisol is stable at room temperature for 2-4 weeks,[64] and alpha-amylase has been shown to be stable under a range of conditions[68]. These procedures for saliva sample collection are routinely used in the field.[64] _ENREF_98 The samples will be stored in our laboratory at -20 °C until they are delivered in a large number to be analyzed at Dresden Lab Service, a well-established biochemical assay laboratory headed by Dr. Kirschbaum, in Germany, to be analyzed. Research personnel will be responsible for the transmission of saliva samples. *The saliva sampling procedures were shown to be* feasible and successful in our study involving 155 Chinese immigrant BCSs, of whom 146 (94.2%) provided at least 14 valid samples of the 16 saliva samples at baseline and 6 weeks later. Participants *did not find the procedure*, including fasting for 1 hour before sampling and mailing saliva samples, *to be burdensome*.

Moderating variables. Acculturation and enculturation will be measured using the Stephenson Multigroup Acculturation Scale.[69] Acculturation toward the dominant American culture will be measured using the Dominant Society Immersion subscale, which has 5 items and good internal reliability (α=0.86),[70,71] *and* enculturation toward the heritage culture will be measured using the Ethnic Society Immersion Subscale, which has 5 items and good internal reliability (α= 0.9).[70,72] Furthermore, potential moderators such as time of diagnosis, age, age of migration will be self-reported at baseline.

Additional exploratory moderating variables include the following:

- Avoidance will be measured using avoidance subscale of the Impact of Event Scale (IES)[73], which has good internal reliability (α=0.77).

- Maintain Harmony will be measured using the maintain harmony subscale of the Brief Collectivism Questionnaire,[74] which was developed to assess general collectivism in Asian culture, while capturing its diverse attitudinal and behavioral manifestations. It has 3 items and good internal reliability (α=0.73).
- Collectivism and emotional control will be measured using the collectivism (3 items) and emotional control (3 items) subscales of the Asian American Values scale, which has good internal reliabilities (α=0.82-0.92 for collectivism subscale and α=.79 - .84).[75]
- Ambivalence over emotional expression will be measured using the Ambivalence over Emotional Expressivity Questionnaire, which has 5 items and good reliability (α=0.80).[76]

Mediating variables.

- Posttraumatic growth will be measured using the Posttraumatic Growth Inventory,[77] which assesses perceived positive changes as a result of a specific traumatic event and has good internal reliability (α=0.93)[77] and validity in Chinese cancer survivors.[78]
- Self-stigma will be measured using a modified version of the 4-item Self-Stigma Scale-Short Form[79] with good reliability ($\alpha \geq 0.90$). It assesses the internalized prejudices and negative feelings individuals have toward themselves due to having breast cancer.
- Harmony will be measured using self-developed harmony scale, which assess participants' perceived agreement of ideas, feelings, and actions with other. It consists of 4 subscales: affective (3 items), behavioral (2 items), cognitive (2 items), and interpersonal relationships (5 items).
- Gratitude will be measured using Gratitude Questionnaire – Six Item Form (GQ-6),[80] which has six items that measure span, frequency, intensity and density of gratitude and primarily focuses on the unidimensional emotional component of gratitude. It has good internal reliability (α=0.87).

Additional exploratory mediating variables including the following:

- Cognitive Appraisal scale will be used to measure how participants appraise the highly stressful events they have experienced, which has 13 items and good internal reliability (α =0.92).[81]
- Self-Compassion will be measured using the self-kindness subscale (5 items) and the mindfulness subscale (2 items) of the Self-Compassion Scale, which has good internal reliability (α =0.86).[82]
- Loneliness will be measured using the 3-item version of University of California, Los Angeles (UCLA)-Loneliness Scale, which has good internal reliability (α =0.88).[83]
- Cancer-related identity will be measured using a question, "When you think about yourself in relation to your cancer, how much does each of these phrases describe you?" (1) a victim of cancer, (2) a cancer patient, (3) a person who has had cancer, (4) a survivor, and (5) a person who recovered, each rated from 1 (not at all) to 5 (Very much).[84]
- Intrusive Thoughts will be measured using the intrusion subscale of the Impact of Event Scale (IES)-6, which has 2 items and good internal reliability (α =0.80).[85]

Mood check

- Global Mood and Life Satisfaction Sliders. Mood will be measured with the Global Mood and Life Satisfaction Sliders.[86] Participants are asked to rate their general affect ("How do you feel right now?") and life satisfaction (How satisfied with your life are you right now?"). The scores range from 0-100 with a higher score indicating greater satisfaction.

Writing Manipulation Check

- Writing manipulation will be measured using 4 questions, “Overall, how personal were the essays that you wrote?”, “Overall, how much did you reveal your emotions in what you wrote?”, “Other than receiving monetary compensation, to what degree has this study been valuable or meaningful for you?”, and “During the writing session, did you gain any new perspective of the disease.” The scores range from 0 (not personal/not at all) to 6 (personal/a lot).
- Writing method (for participants who use REDCap for the writing tasks) will be assessed using two items, “Which method did you use for the writing tasks?” (1 = typing, 2 voice-to-text, 3. both typing and voice-to-text) and (answer if participant select 2 or 3) “In which writing session(s) do you use voice-to-text, or both typing and voice-to-text?” (Allows multiple choices; 1 = 1st writing task, 2 = 2nd writing task, 3 = 3rd writing task)

7.0 Data and Specimen Banking

Power analysis. Power analysis is based on testing hypotheses 1.1 in Aim 1 (primary aim) for the primary endpoints (QOL evaluated at 6 and 12 months) using nQuery 7.0. A sample size of 64 in each group (80 original, 20% attrition at 12 months, an assumption based on preliminary study 2) will have at least *80%* power to detect a difference in mean QOL of 0.60 standard deviation (SD) using a two-sample *t*-test with a two-sided ***0.0125*** *significance level (with a Bonferroni adjustment for the four primary comparisons).* The t-test is appropriate to estimate power because the primary hypothesis is specified as contrasts at specific time points. The effect size of 0.60 was estimated from preliminary study 2,[46] which compared expressive writing with the control at 6 months. For the same reason, we expect that the self-cultivation intervention condition will have an effect size of 0.6 at the follow-ups. The same sample size will have 80% power to detect a standardized effect size of 0.499 (a medium effect size) in between-group differences in cortisol slopes and perceived stress (hypothesis 1.2), to detect a standardized effect size of 0.251 of the interaction between acculturation and treatment conditions (hypothesis 2.1), and to detect a mediation effect (standardized coefficient of 0.35 in the b path and standardized between-group difference of 0.60) (hypothesis 3.1), with a 0.05 two-sided significance level.

Quantitative data analysis. Before we begin inferential procedures, we will conduct extensive descriptive analyses on the data collected. Distributional assumptions will be evaluated, and if indicated, normalizing (such as log) transformations or robust procedures will be used. Appropriate procedures to assess the validity and reliability of questionnaires will be used.

Aim 1. Given the longitudinal nature of the study, we will use linear mixed-effects models (LMMs) to assess the effects of the interventions on outcomes. Treatment condition and time will be included and tested in the model, as well as their interaction, with adjustment for covariates[87] including the baseline outcome value, time since diagnosis, and disease stage. If needed, we will also control for potential confounders such as demographic or other clinical variables correlated with outcome variables. Potential heterogeneity across recruitment sites will be accounted for by adding site-specific random intercepts. The repeated-measures correlation structure will be selected based on the Bayesian information criterion (BIC). The primary outcome will be a composite scale of QOL based on the FACT-B total score. Hypothesis 1.1 (QOL at 6- and 12-month follow-ups for both intervention conditions) will be tested against the control condition within the LMMs based on appropriately constructed contrasts, each with a *two-sided 0.0125 level of significance, to ensure that the overall type I error rate across the four tests does not exceed 0.05*. *Controlling the overall type I error rate for testing the four primary hypotheses will help* ***increase the reproducibility*** *of our main study findings.*

All other analyses described below will use a two-sided significance level of 0.05 without adjustment for multiple testing. The corresponding results will thus be interpreted as hypothesis-

generating. The above LMMs will be repeated for medical appointments and perceived stress as outcomes (hypotheses 1.1 and 1.2). We will conduct linear regression analyses with stress biomarkers (cortisol and alpha-amylase) as the outcomes (hypothesis 1.2). The average levels and average slope of change in the 2 days at the 6-week follow-up will be used as the outcome of interest for each stress biomarker.[88,89] We will control for potential confounding variables such as medical conditions and medications.

Aim 2. To characterize the moderation effect of acculturation/enculturation on the intervention (hypothesis 2.1), we will use LMMs like those in Aim 1 but test additional interaction effects between acculturation/enculturation subscale composite scores and intervention conditions. Items to assess the language aspects in the acculturation/enculturation subscales will also be explored as potential moderators. Furthermore, we will test interaction effects between intervention conditions and additional potential moderators (time since diagnosis, age, age of migration, , intervention delivery modes (receiving instructions via mail and writing by hand vs. receiving instructions and writing online), because the effects of the interventions may also depend on these factors. For example, those who are more recently diagnosed or more comfortable writing in Chinese may benefit more from the interventions.

Aim 3. To identify potential mechanisms by which the expressive writing intervention yields health benefits (hypothesis 3.1), we will conduct a longitudinal mediation analysis.[90] Specifically, we will use measures of QOL outcomes at 6- and 12-month follow-ups and each of the potential mediators (posttraumatic growth, relationship harmony, and self-stigma) at 6-week and 6-month follow-ups, respectively, in the a and/or b paths of the mediation models (each path being a LMM[90]). We will use bootstrapping in testing for the indirect effect of each mediator on QOL outcome.[91] We will also explore multiple mediator models in case more than one mediator emerges.[92]

Missing data and dropouts. The LMMs, as a likelihood-based regression method, will give valid (e.g., asymptotically unbiased) estimates of effects provided that the missingness probability depends only on the observed variables in the model (missing at random). However, data may not be missing at random; thus, we will conduct sensitivity analyses assuming different missing data mechanisms, especially when the attrition rate is moderate or high (e.g., >10%) and/or the attrition is unbalanced across intervention conditions. We will consider multiple imputation approaches based on relevant baseline participant characteristics to account for the missing-at-random mechanisms. We will also explore pattern-mixture and selection models to account for potential missing-not-at-random mechanisms.[93]

Linguistic and Content Analyses (Aim 3 Only)

The linguistic and content analyses aim to provide a nuanced understanding of the “how” and “why” of intervention processes within a cultural context. These analyses have the advantage of revealing cultural concepts and culturally specific phenomena, which may improve the explanatory power of the existing psychosocial theories and hypothesized mediators.

Linguistic analysis. With guidance from linguistic analysis expert Dr. Pennebaker, we will conduct linguistic analysis to reveal differences in language use among conditions. We will follow procedures from our previous studies[51] and analyze the written essays using Linguistic Inquiry and Word Count (LIWC) software.[53] Self-regulation assumes that individuals work through thoughts and emotions in an attempt to reappraise past stressful experiences.[94] This process requires the use of self-references, negative emotion words, and past-tense verbs. In contrast, self-cultivation involves a focus on the surrounding relational and social environment and therefore should spur the use of greater social and other-oriented words and relatively fewer self-references.[95] We will use inductive topic modeling analytic methods, including the Meaning Extraction Method,[96,97] a factor analytically driven method. Such a method should provide the frequency of the most-used words and identify differences in the content themes that each type of writing encourage. Chinese

versions of the LIWC computer program are available. Dr. Pennebaker has also developed a Chinese tokenizer that allows the use of the Meaning Extraction Method in Chinese. *Triangulated data analysis.* Regression analyses will be performed to evaluate the effects of the linguistic features of the essays (such as the frequency of positive emotional words used in the essays) on the improvement of QOL.

Content analysis. The content analysis complements the quantitative and linguistic analyses *by providing a detailed narrative contextual and cultural account of the findings* revealed in quantitative and linguistic analyses. The PI and the expert in deductive qualitative and mixed-method studies, Dr. Kagawa-Singer, will develop a codebook with guidelines and codes for analyzing written essays. According to the grounded theory approach, the constant comparative method will be used to develop a basic group of codes that inductively emerge from the data through intensive reading of the material, comparison and differentiation of statements, and development of descriptive categories showing the relationships of codes.[98,99] A coding tree with the key categories will be developed to describe the relationships of codes as they emerge from the data. We will look for the unique and culturally specific items, identify differences in the content themes among the three conditions, and develop an integrated understanding of the effects of the intervention on the breast cancer experience. The team has experience analyzing written essays.[31,32] *Triangulated data analysis.* We will use the content analysis above to provide cultural context to augment interpretation of quantitative data analysis of hypothesized mediators. We will also integrate code frequencies indicative of culturally relevant constructs into quantitative data, and then follow the mediation analysis (aim 3-quantitive data analysis) to test these additional cultural factors as potential mediators to explain the benefits of the intervention.

Data and specimen banking. Participants will provide saliva samples for this study to test cortisol and alpha-amylase*, two* objective measures of stress *that* will provide a window into the biological impacts of the intervention. All saliva samples will be assigned a unique identifier and will be stored securely at -20°C at MD Anderson Cancer Center, for a minimum of 3 years and maximum of 7 years after the completion of the study. Data from the analysis of the saliva will be entered into a computer and will not be associated with personal identifiers. No information on individual participants will be released, and only quantitative and qualitative summaries will be reported. Only the PI and trained research staff involved in the project will have access to the samples. All saliva samples will be destroyed in accordance with policy at the end of 7 years after the study is completed.

8.0 Sharing of Results with Subjects

Individual subject results will not be shared with participants or others.

9.0 Study Timelines*

The duration of an individual subject's participation in the study is 12 months. It is anticipated that enrollment will last for 4 years and the study will be completed in 5 years.

10.0 Inclusion and Exclusion Criteria*

To be included in the study, participants must satisfy the following eligibility criteria:

1. Being diagnosed with stage 0-III breast cancer.
2. Completed primary treatment, including surgery, chemotherapy, radiation therapy, immunotherapy, and/or targeted therapy within the preceding 5 years.
4. Foreign-born Chinese woman (aged 18 and older) who lived in the United States for at least 6 months in the past.

Exclusion criteria include inability to provide informed consent.

11.0 Vulnerable Populations

N/A

12.0 Local Number of Subjects

We expect to recruit a total sample of 240 participants across the nation.

13.0 Recruitment Methods

We will identify potential participants by working with three community organizations and

Community organizations serving Chinese patients. We will recruit primarily through three community partners: 1) Herald Cancer Association (HCA) and its local offices in Southern California and New York, 2) Shanti Project organization in San Francisco, and 3) the Light and Salt Association in Houston. Through community outreach, these organizations are well-connected with local Chinese immigrant BCSs.

Our success in recruitment for previous studies supports our ability to recruit for the proposed study. Our community partners at HCA (Los Angeles, San Diego, and New York), Shanti Project in San Francisco, and Light and Salt Association in Houston have experience recruiting Chinese breast cancer survivors for large funded studies. They are confident they will be able to work with us to recruit from Southern California and New York, San Francisco, and Houston and to reach the recruitment goal (N=240). Our team's experience and cultural and linguistic compatibility with the targeted population uniquely position us to achieve the recruitment goal.

Advertising in the Chinese community. Advertising in social media in the Chinese community will be particularly helpful to recruit isolated Chinese immigrant BCSs who are not connected to community-based organizations. IRB-approved advertisements will be placed in popular Chinese media and where Chinese immigrants congregate (e.g., community centers, churches, cultural events, and popular supermarkets).

Participants will receive up to $200 as reasonable compensation for finishing the study (A total of $50 for all saliva samples provided for the period of the study; $45 for the three essays; and $25, $15, $25 and $25 for the questionnaires at baseline, 6-week, 6-month and 12-month, respectively. A bonus of $15 will be provided for the completion of the study) and prorated compensation if they withdraw. Small study-related gifts will be provided to everyone enrolled.

Recruitment from within MD Anderson. We will also recruit through the University of Texas MD Anderson Cancer Center to supplement any shortfall in accrual through the community partners. Patients eligible for inclusion will be identified through reports generated from MD Anderson patient databases. Texas and California cancer registries will be utilized if needed.

14.0 Withdrawal of Subjects

All participation will be with fully informed consent, and participants are free to withdraw from the study at any time without penalty. Reasons for withdrawal will be documented. These analyses will help inform conclusions about the generalizability of the study.

15.0 Risks to Subjects

The possible risks/discomforts for participants associated with this research are minimal, and may only include transitory feelings of emotional discomfort associated with answering questions about their breast cancer experience. Although some participants may find reflecting on their cancer experience to be upsetting, this distress is likely to be no more than encountered in the daily lives of these breast cancer survivors.

16.0 Potential Benefits to Subjects

The proposed intervention may benefit participants' mental and physical health.

17.0 Data Management and Confidentiality

All research personnel will be experienced researchers who are culturally competent and have completed all required human subjects research training. The PI will develop quality assurance procedures for data management. Study investigators and staff will maintain quality assurance procedures for all data collected. Measures to ensure quality control of collected data include pre-entry review by data management staff, review of entered data against scanned paper copies, frequent data audits that involve randomly selecting a sample of data and comparing it against the entered data, using aggregate reports to spot errors, and developing a data dictionary to systematically catalog and communicate the structure and content of the data.

Commented [L1]: Staff at community sites do not have access to our data.

Study materials delivered by mail will be sent by the research team at The University of Texas MD Anderson Cancer Center. Participants who opt for the mail-in method will mail their questionnaires packet and essays completed via paper/pen directly to the research team at MD Anderson via prepaid envelopes, they can also choose to email scanned copies to the research team. Those who choose to complete the study electronically will be completing questionnaire assessments and writing tasks via personalized REDCap survey links. Paper copies of study materials used in the recruitment process at the community partner sites will be scanned and stored in a secure server and mailed to the MD Anderson research team. All paper questionnaires packets and essays will be stored in a secure and locked location at UT MD Anderson Cancer Center under the supervision of Dr. Qian Lu's research team. All protected health information (PHI) records obtained will be kept in accordance with relevant HIPAA requirements. Participants' quantitative assessments, essays and saliva samples will be coded only by participant ID numbers. The keys linking names and addresses to code numbers will be kept in a secure and locked location that is separate from the location where the data are stored. Only key research personnel will have access to the information. All computer files will also be password-protected and stored on MDACC servers behind the firewall. These files will only be accessed through MDACC machines by individuals with the appropriate permissions. All PHI will be removed from the data when it is exported for analysis. Data will be cross-checked to ensure accuracy.

18.0 Provisions to Monitor the Data to Ensure the Safety of Subjects

N/A.

19.0 Provisions to Protect the Privacy Interests of Subjects

No information on individual participants will be disclosed, and only quantitative and qualitative summaries will be reported. Participants' quantitative assessments, essays and saliva samples will be coded by participant ID numbers, only. The keys linking names and addresses to code numbers will be kept in a secure and locked location that is separate from the location where the data are stored. Only key research personnel will have access to the information. All computer files will be password-protected.

20.0 Compensation for Research-Related Injury

N/A – The study includes minimal risks to participants.

21.0 Economic Burden to Subjects

There is no study-related cost that participants will be responsible for covering. Participants will be asked to provide consent to receiving SMS messages if they choose to use text messages as the communication method.

22.0 Consent Process

Participation in the study will require fully informed consent. All participants will be provided with a detailed verbal and/or written description including 1) brief description of the research 2) protections for confidentiality (e.g., answers will only be seen by project staff); 3) and rights as a

participant in human subjects research (e.g., participation is voluntary). Consent documents will be in Chinese. English documents will be provided upon request. Personnel interacting with potential participants will be experienced researchers or community workers fluent in Chinese (Mandarin and/or Cantonese) and English. All participants will be told they have the option to decline study participation, withdraw at any time or refuse to complete any section of the study that makes them feel uncomfortable without fear of repercussion or penalty.

Participants will be consented through contact with the research team. Research staff at The University of Texas MD Anderson Cancer Center and/or the community sites may conduct the screening process by asking for the participants' contact information and reaching out to potential participants to confirm eligibility, ask their language preference, and describe to them all data that will be collected and how they will be collected. Participants will be encouraged and given the opportunity to ask questions and seek clarifications. Demographic details such as birth date will also be collected for subject registration purposes. Participants consenting to the study will sign the informed consent and will be provided a copy of the signed consent form. By consenting to be in the study, the participants grant the research team permission to access and use their information as outlined in the consent form.

23.0 Setting

The study will be led by researchers in the Department of Health Disparities Research (HDR) at The University of Texas MD Anderson Cancer Center. The Department of Health Disparities Research has ample office space housing all faculty and support staff. The space includes common work/meeting areas, and conference rooms. The Principal Investigator, co-investigators, and support staff all have adequate office space, equipment and administrative support to carry out the project. This includes access to telephones and telephone airtime and file drawers to store any hard copies of documents. All faculty and research staff in the Departments of Health Disparities are provided with the necessary hardware and software for general word processing, statistical analyses, and database applications.

We will recruit through community partners: the HCA and its local chapters in Southern California (Los Angeles,San Diego, and Orange County) and New York, the Light and Salt organization in Houston, and Shanti Project in San Francisco. The role of the community partners is to assist MDA researchers with various study activities including recruiting participants, addressing questions or concerns participants may have, and helping to retain participants. None of the community sites has an IRB; therefore, all the community sites will rely on MD Anderson as the IRB on record.

Community staff have extensive experience working on funded research projects focused on Asian/Chinese American breast cancer survivors. HCA staff have served on several NIH-funded research projects collaborating with academic institutions and cancer centers. HCA has successfully collaborated with the PI, Dr. Qian Lu, to complete protocols for three large studies, including 2 R01s, one of which was a randomized controlled trial investigating the effectiveness of a social support program on Chinese breast cancer survivors. That study, which was funded by the NIH, included longitudinal data collection and self-reported and biological measures. HCA's specific tasks on the prior projects included recruitment and enrollment, data collection, volunteer training and focus group facilitation.

Community partners have completed the appropriate regulatory trainings including the protection of human subjects in research. Additional protocol- and institution-specific trainings related to policies and procedures will be conducted by the research team, including training on how to obtain and document consent in accordance with MD Anderson standard operating procedures (SOPs). Partners will also be provided with the investigator manual (HRP 103) and the human research protection program plan (HRP 101) for review and to use as reference to guide research activities. MD Anderson researchers will provide oversight of the partner sites and

researchers to ensure that the research is conducted per protocol and MDA policies and procedures governing research are being observed.

The study will be guided by a community advisory board composed of at least five leaders from the community organizations. The board will meet quarterly and will provide input on recruitment and retention and overall study design and implementation.

24.0 Resources Available

There are adequate resources available to recruit 240 participants in a 3-year period and complete the study in the proposed timeline. Our community partners are well-connected with local Chinese immigrant BCSs. All research staff participating in the study will be fully trained about protection of human research subjects, the protocol, the research procedures, and their duties and functions.

References

1. National Cancer Institute. Cancer statistics. National Cancer Institute. Understanding cancer Web site.
2. National Cancer Institute. Financial burden of cancer care. National Cancer Institute. Life after cancer Web site.
3. Brown ML, Lipscomb J, Snyder C. The burden of illness of cancer: Economic cost and quality of life. *Annual Review of Public Health.* 2001;22(1):91-113.
4. Ferrell BR, Hassey Dow K, Grant M. Measurement of the quality of life in cancer survivors. *Quality of Life Research.* 1995;4(6):523-531.
5. Montazeri A. Quality of life data as prognostic indicators of survival in cancer patients: an overview of the literature from 1982 to 2008. *Health and Quality of Life Outcomes.* 2009;7(1):102.
6. Epplein M, Zheng Y, Zheng W, et al. Quality of life after breast cancer diagnosis and survival. *Journal of Clinical Oncology.* 2011;29(4):406-412.
7. Rendas-Baum R, D'Alessio D, Bjorner JB. Health-related quality of life predicted subsequent health care resource utilization in patients with active cancer. *Quality of Life Research.* 2019;28(4):1085-1095.
8. Seid M, Varni JW, Segall D, Kurtin PS. Health-related quality of life as a predictor of pediatric healthcare costs: a two-year prospective cohort analysis. *Health and quality of life outcomes.* 2004;2:48-48.
9. Chida Y, Hamer M, Wardle J, Steptoe A. Do stress-related psychosocial factors contribute to cancer incidence and survival? *Nature Clinical Practice Oncology.* 2008;5:466.
10. Allen JD, Savadatti S, Gurmankin Levy A. The transition from breast cancer 'patient' to 'survivor'. *Psycho-Oncology.* 2009;18(1):71-78.
11. Ganz PA, Coscarelli A, Fred C, Kahn B, Polinsky M, Petersen L. Breast cancer survivors: Psychosocial concerns and quality of life. *Breast Cancer Research and Treatment.* 1996;38(2):183-199.
12. Maher J, Fenlon D. The psychosocial issues of survivorship in breast cancer. *Advances in Breast Cancer.* 2010;7(2):17-22.
13. Ell KO, Mantell JE, Hamovitch MB, Nishimoto RH. Social support, sense of control, and coping among patients with breast, lung, or colorectal cancer. *Journal of Psychosocial Oncology.* 1989;7(3):63-89.
14. Hewitt M, Herdman R, Holland J. Meeting the psychosocial needs of women with breast cancer In. Washington: DC National Academies Press; 2004.
15. Andersen BL, Yang HC, Farrar WB, et al. Psychologic intervention improves survival for breast cancer patients: a randomized clinical trial. *Cancer.* 2008;113(12):3450-3458.
16. Stagl JM, Lechner SC, Carver CS, et al. A randomized controlled trial of cognitive-behavioral stress management in breast cancer: survival and recurrence at 11-year follow-up. *Breast Cancer Research and Treatment.* 2015;154(2):319-328.
17. Ashing-Giwa KT, Tejero JS, Kim J, Padilla GV, Hellemann G. Examining predictive models of HRQOL in a population-based, multiethnic sample of women with breast carcinoma. *Quality of Life Research.* 2007;16(3):413-428.
18. Janz NK, Mujahid MS, Hawley ST, et al. Racial/ethnic differences in quality of life after diagnosis of breast cancer. *Journal of Cancer Survivorship.* 2009;3(4):212-222.

19. Eversley R, Estrin D, Dibble S, Wardlaw L, Pedrosa M, Favila-Penney W. Post-treatment symptoms among ethnic minority breast cancer survivors. *Oncology Nursing Forum.* 2005;32(2):250-256.
20. Pew Research Center. *The rise of asian americans.* pewresearch.org Jun 19 2012.
21. Torre LA, Sauer AM, Chen MSJ, Kagawa-Singer M, Jemal A, Siegel RL. Cancer statistics for Asian Americans, Native Hawaiians, and Pacific Islanders, 2016: converging incidence in males and females. *CA: a Cancer Journal for Clinicians.* 2016;66(3):182-202.
22. Gomez SL, Quach T, Horn-Ross PL, et al. Hidden breast cancer disparities in Asian women: disaggregating incidence rates by ethnicity and migrant status. *American Journal of Public Health.* 2010;100(Supplement 1):S125-S131.
23. Hoeffel EM, Rastogi S, Kim MO, Shahid H. *The Asian population: 2010.* U.S. Census Bureau; Mar 2012.
24. Lopez G, Ruiz NG, Patten E. Key facts about Asian Americans, a diverse and growing population. Pew Research Center. Fact Tank Web site.
25. Lopez G, Cilluffo A, Patten E. Chinese in the U.S. Fact Sheet. Pew Research Center. Pew Research Center: Social & Demographic Trends Web site.
26. Gomez SL, Clarke CA, Shema SJ, Chang ET, Keegan TH, Glaser SL. Disparities in breast cancer survival among Asian women by ethnicity and immigrant status: a population-based study. *American Journal of Public Health.* 2010;100(5):861-869.
27. Kagawa-Singer M, Lu Q, Valdez Dadia A. *Culture, social support & quality of life: Asian American breast cancer survivors.* October 31 2017. 5R01CA158314-05.
28. Kagawa-Singer M, Wellisch D, Durvasula R. Impact of breast cancer on Asian American and Anglo American women. *Cult Med Psychiatry.* 1997;21(4):449-480.
29. Kagawa-Singer M, Wellisch DK. Breast cancer patients' perceptions of their husbands' support in a cross-cultural context. *Psycho-Oncology.* 2003;12(1):24-37.
30. Lee S, Chen L, Ma GX, Fang CY, Oh Y, Scully L. Challenges and needs of Chinese and Korean American breast cancer survivors: in-depth interviews. *North American Journal of Medicine & Science.* 2013;6(1):1-8.
31. Lu Q, Yeung NC, You J, Dai J. Using expressive writing to explore thoughts and beliefs about cancer and treatment among Chinese American immigrant breast cancer survivors. *Psycho-Oncology.* 2016;25(11):1371-1374.
32. Warmoth K, Cheung B, You J, Yeung NCY, Lu Q. Exploring the social needs and challenges of Chinese American immigrant breast cancer survivors: a qualitative study using an expressive writing approach. *Int J Behav Med.* 2017;24(6):827-835.
33. Wong-Kim E, Sun A, Merighi JR, Chow EA. Understanding quality-of-life issues in Chinese women with breast cancer: a qualitative investigation. *Cancer Control: Cancer, Culture, and Literacy Supplement.* 2005;12(Supplement 2):6-12.
34. Chen GM, Chung J. The impact of Confucianism on organizational communication. *Communication Quarterly.* 1994;42(2):93-105.
35. U.S. Department of Health and Human Services. Profile: Asian Americans. U.S. Department of Health and Human Services.
36. Lu Q, Zheng D, Young L, Kagawa-Singer M, Loh A. A pilot study of expressive writing intervention among Chinese-speaking breast cancer survivors. *Health Psychology.* 2012;31(5):548-551.

37. Pennebaker JW, Beall SK. Confronting a traumatic event: toward an understanding of inhibition and disease. *Journal of Abnormal Psychology.* 1986;95(3):274-281.
38. Frattaroli J. Experimental disclosure and its moderators: a meta-analysis. *Psychol Bull.* 2006;132(6):823-865.
39. Pennebaker JW. Writing about emotional experiences as a therapeutic process. *Psychological Science.* 1997;8(3):162-166.
40. Smyth JM. Written emotional expression: effect sizes, outcome types, and moderating variables. *Journal of Consulting and Clinical Psychology.* 1998;66(1):174-184.
41. Merz EL, Fox RS, Malcarne VL. Expressive writing interventions in cancer patients: a systematic review. *Health Psychology Review.* 2014;8(3):339-361.
42. Stanton AL, Danoff-Burg S, Sworowski LA, et al. Randomized, controlled trial of written emotional expression and benefit finding in breast cancer patients. *Journal of Clinical Oncology.* 2002;20(20):4160-4168.
43. Henry EA, Schlegel RJ, Talley AE, Molix LA, Bettencourt BA. The feasibility and effectiveness of expressive writing for rural and urban breast cancer survivors. *Oncology Nursing Forum.* 2010;37(6):749-757.
44. Gellaitry G, Peters K, Bloomfield D, Horne R. Narrowing the gap: the effects of an expressive writing intervention on perceptions of actual and ideal emotional support in women who have completed treatment for early stage breast cancer. *Psycho-Oncology.* 2010;19(1):77-84.
45. Kagawa Singer M, Dressler W, George S, Elwood W. *The NIH Expert Panel. The cultural framework for health: an integrative approach for research and program design and evaluation.* 2015.
46. Lu Q, Gallagher M, Young L, Loh A. Expressive writing intervention improves quality of life among Chinese-American breast cancer survivors: a randomized controlled trial. *Annals of Behavioral Medicine.* 2018;52(11):952-962.
47. Perry M, Faes M, Reelick MF, Rikkert MGMO, Borm GF. Studywise minimization: a treatment allocation method that improves balance among treatment groups and makes allocation unpredictable. *Journal of Clinical Epidemiology.* 2010;63(10):1118-1122.
48. Pocock SJ. *Clinical trials: a practical approach.* John Wiley & Sons Ltd.; 2013.
49. Pocock SJ, Simon R. Sequential treatment assignment with balancing for prognostic factors in the controlled clinical trial. *Biometrics.* 1975;31(1):103-115.
50. The University of Texas M.D. Anderson Cancer Center Department of Biostatistics. The Clinical Trial Web Application. The University of Texas M.D. Anderson Cancer Center. Accessed.
51. Lu Q, Wong CC, Gallagher MW, Tou RY, Young L, Loh A. Expressive writing among Chinese American breast cancer survivors: a randomized controlled trial. *Health Psychology.* 2017;36(4):370-379.
52. Pennebaker JW, Colder M, Sharp LK. Accelerating the coping process. *Journal of Personality and Social Psychology.* 1990;58(3):528-537.
53. Pennebaker JW, Boyd RL, Jordan K, Blackburn K. *The development and psychometric properties of LIWC2015.* Austin, TX: University of Texas at Austin;2015.
54. Ryan R, Booth S, Spathis A, Mollart S, Clow A. Use of salivary diurnal cortisol as an outcome measure in randomised controlled trials: A systematic review. *Annals of Behavioral Medicine.* 2016;50(2):210-236.

55. Brady MJ, Cella DF, Mo F, et al. Reliability and validity of the functional assessment of cancer therapy-breast quality-of-life instrument. *Journal of Clinical Oncology.* 1997;15(3):974-986.
56. Wan C, Zhang D, Yang Z, et al. Validation of the simplified Chinese version of the FACT-B for measuring quality of life for patients with breast cancer. *Breast Cancer Research and Treatment.* 2007;106(3):413-418.
57. You J, Lu Q. Social constraints and quality of life among Chinese-speaking breast cancer survivors: a mediation model. *Quality of Life Research.* 2014;23(9):2577-2584.
58. Cohen S, Kamarck T, Mermelstein R. A global measure of perceived stress. *Journal of Health and Social Behavior.* 1983;24(4):385-396.
59. Simard S, Thewes B, Humphris G, et al. Fear of cancer recurrence in adult cancer survivors: a systematic review of quantitative studies. *J Cancer Surviv.* 2013;7(3):300-322.
60. Bredle JM, Salsman JM, Debb SM, Arnold BJ, Cella D. Spiritual Well-Being as a Component of Health-Related Quality of Life: The Functional Assessment of Chronic Illness Therapy—Spiritual Well-Being Scale (FACIT-Sp). *Religions.* 2011;2(1):77-94.
61. Buysse DJ, Reynolds III CF, Monk TH, Berman SR, Kupfer DJ. The Pittsburgh Sleep Quality Index: a new instrument for psychiatric practice and research. *Psychiatry research.* 1989;28(2):193-213.
62. Cella D, Choi SW, Condon DM, et al. PROMIS(®) Adult Health Profiles: Efficient Short-Form Measures of Seven Health Domains. *Value Health.* 2019;22(5):537-544.
63. Sephton SE, Sapolsky RM, Kraemer HC, Spiegel D. Diurnal cortisol rhythm as a predictor of breast cancer survival. *Journal of the National Cancer Institute.* 2000;92(12):994-1000.
64. Kirschbaum C, Hellhammer DH. Salivary cortisol in psychoneuroendocrine research: Recent developments and applications. *Psychoneuroendocrinology.* 1994;19(4):313-333.
65. AARDEX Group. MEMS® Cap versatile adherence monitoring cap. AARDEX Group. Accessed.
66. Kudielka BM, Broderick JE, Kirschbaum C. Compliance with saliva sampling protocols: electronic monitoring reveals invalid cortisol daytime profiles in noncompliant subjects. *Psychosomatic Medicine.* 2003;65(2):313-319.
67. Broderick JE, Arnold D, Kudielka BM, Kirschbaum C. Salivary cortisol sampling compliance: comparison of patients and healthy volunteers. *Psychoneuroendocrinology.* 2004;29(5):636-650.
68. O'Donnell K, Kammerer M, O'Reilly R, Taylor A, Glover V. Salivary alpha-amylase stability, diurnal profile and lack of response to the cold hand test in young women. *Stress (Amsterdam, Netherlands).* 2009;12(6):549-554.
69. Stephenson M. Development and validation of the Stephenson Multigroup Acculturation Scale (SMAS). *Psychological Assessment.* 2000;12(1):77-88.
70. Chu Q, Wong CCY, Lu Q. Acculturation moderates the effects of expressive writing on post-traumatic stress symptoms among Chinese American breast cancer survivors. *Int J Behav Med.* 2019;26(2):185-194.
71. Lau AS, Fung JJ, Ho LY, Liu LL, Gudiño OG. Parent training with high-risk immigrant Chinese families: A pilot group randomized trial yielding practice-based evidence. *Behavior Therapy.* 2011;42(3):413-426.
72. Abad NS, Sheldon KM. Parental autonomy support and ethnic culture identification among second-generation immigrants. *Journal of Family Psychology.* 2008;22(4):652.

73. Horowitz M, Wilner N, Alvarez W. Impact of Event Scale: a measure of subjective stress. *Psychosom Med.* 1979;41(3):209-218.
74. Lui PP, Rollock D. Greater than the sum of its parts: Development of a measure of collectivism among Asians. *Cultur Divers Ethnic Minor Psychol.* 2018;24(2):242-259.
75. Kim BK, Li LC, Ng GF. The Asian American values scale--multidimensional: development, reliability, and validity. *Cultur Divers Ethnic Minor Psychol.* 2005;11(3):187-201.
76. King LA, Emmons RA. Conflict over emotional expression: psychological and physical correlates. *J Pers Soc Psychol.* 1990;58(5):864-877.
77. Tedeschi RG, Calhoun LG. The posttraumatic growth inventory: measuring the positive legacy of trauma. *Journal of Traumatic Stress.* 1996;9(3):455-471.
78. Ho SM, Chan CL, Ho RT. Posttraumatic growth in Chinese cancer survivors. *Psycho-Oncology.* 2004;13(6):377-389.
79. Yeung NCY, Lu Q, Mak WWS. Self-perceived burden mediates the relationship between self-stigma and quality of life among Chinese American breast cancer survivors. *Support Care Cancer.* 2019;27(9):3337-3345.
80. McCullough ME, Emmons RA, Tsang J-A. The grateful disposition: A conceptual and empirical topography. *Journal of Personality and Social Psychology.* 2002;82(1):112-127.
81. Stanton AL, Tennen H, Affleck G, Mendola R. Cognitive appraisal and adjustment to infertility. *Women Health.* 1991;17(3):1-15.
82. Neff KD. The development and validation of a scale to measure self-compassion. *Self and Identity.* 2003;2(3):223-250.
83. Hughes ME, Waite LJ, Hawkley LC, Cacioppo JT. A Short Scale for Measuring Loneliness in Large Surveys: Results From Two Population-Based Studies. *Res Aging.* 2004;26(6):655-672.
84. Park CL, Zlateva I, Blank TO. Self-identity after cancer: "survivor", "victim", "patient", and "person with cancer". *J Gen Intern Med.* 2009;24 Suppl 2(Suppl 2):S430-435.
85. Thoresen S, Tambs K, Hussain A, Heir T, Johansen VA, Bisson JI. Brief measure of posttraumatic stress reactions: impact of Event Scale-6. *Soc Psychiatry Psychiatr Epidemiol.* 2010;45(3):405-412.
86. Bao KJ. The course of well-being in romantic relationships: Predicting positive affect in dating participants. *Psychology.* 2012;3(12):1091.
87. Kahan BC, Morris TP. Improper analysis of trials randomised using stratified blocks or minimisation. *Statistics in Medicine.* 2012;31(4):328-340.
88. Lipschitz DL, Kuhn R, Kinney AY, Donaldson GW, Nakamura Y. Reduction in salivary α-amylase levels following a mind-body intervention in cancer survivors: an exploratory study. *Psychoneuroendocrinology.* 2013;38(9):1521-1531.
89. Nater UM, Rohleder N, Schlotz W, Ehlert U, Kirschbaum C. Determinants of the diurnal course of salivary alpha-amylase. *Psychoneuroendocrinology.* 2007;32(4):392-401.
90. MacKinnon DP. *Introduction to statistical mediation analysis.* 1st ed. New York: Routledge; 2008.
91. Preacher KJ, Hayes AF. SPSS and SAS procedures for estimating indirect effects in simple mediation models. *Behavior Research Methods, Instruments, & Computers.* 2004;36(4):717-731.

92. Preacher KJ, Hayes AF. Asymptotic and resampling strategies for assessing and comparing indirect effects in multiple mediator models. *Behavior Research Methods.* 2008;40(3):879-891.
93. Little RJA, Rubin DB. *Statistical analysis with missing data.* Vol 333: John Wiley & Sons, Inc.; 2014.
94. Klein K, Boals A. Coherence and narrative structure in personal accounts of stressful experiences. *Journal of Social and Clinical Psychology.* 2010;29(3):256-280.
95. Tausczik YR, Pennebaker JW. The psychological meaning of words: LIWC and computerized text analysis methods. *Journal of Language and Social Psychology.* 2010;29(1):24-54.
96. Chung C, Pennebaker J. Revealing dimensions of thinking in open-ended self-descriptions: an automated meaning extraction method for natural language. *Journal of Research in Personality.* 2008;42(1):96-132.
97. Kramer ADI, Chung C. Dimensions of self-expression in Facebook status updates. Fifth International AAAI Conference on Weblogs and Social Media; 2011; Barcelona, Catalonia, Spain.
98. Bernard HR. *Research methods in anthropology: qualitative and quantitative approaches.* 4 ed. Oxford, UK: Altamira Press; 2006.
99. Willms DG, Best JA, Taylor DW, et al. A systematic approach for using qualitative methods in primary prevention research. *Medical Anthropology Quarterly.* 1990;4(4):391-409.
